# Type 2 and type 17 effector cells are increased in the duodenal mucosa but not peripheral blood of patients with functional dyspepsia

**DOI:** 10.3389/fimmu.2022.1051632

**Published:** 2023-01-06

**Authors:** Grace L. Burns, Jessica K. Bruce, Kyra Minahan, Andrea Mathe, Thomas Fairlie, Raquel Cameron, Crystal Naudin, Prema M. Nair, Michael D. E. Potter, Mudar Zand Irani, Steven Bollipo, Robert Foster, Lay T. Gan, Ayesha Shah, Natasha A. Koloski, Paul S. Foster, Jay C. Horvat, Martin Veysey, Gerald Holtmann, Nick Powell, Marjorie M. Walker, Nicholas J. Talley, Simon Keely

**Affiliations:** ^1^ College of Health, Medicine and Wellbeing, The University of Newcastle, Callaghan, NSW, Australia; ^2^ Immune Health Research Program, Hunter Medical Research Institute, New Lambton Heights, NSW, Australia; ^3^ National Health & Medical Research Council (NHMRC) Centre of Research Excellence in Digestive Health, University of Newcastle, Newcastle, NSW, Australia; ^4^ Translational Research Institute, Brisbane, QLD, Australia; ^5^ Department of Gastroenterology and Hepatology, Princess Alexandra Hospital, Brisbane, QLD, Australia; ^6^ Department of Gastroenterology, John Hunter Hospital, Newcastle, NSW, Australia; ^7^ Hull-York Medical School, University of Hull, Hull, United Kingdom; ^8^ Faculty of Medicine, Imperial College London, London, United Kingdom

**Keywords:** functional dyspepsia, T cells, lymphocytes, immunology, functional gastrointestinal disorder (FGID)

## Abstract

**Background:**

Functional dyspepsia is characterised by chronic symptoms of post-prandial distress or epigastric pain not associated with defined structural pathology. Increased peripheral gut-homing T cells have been previously identified in patients. To date, it is unknown if these T cells were antigen-experienced, or if a specific phenotype was associated with FD.

**Objective:**

This study aimed to characterise T cell populations in the blood and duodenal mucosa of FD patients that may be implicated in disease pathophysiology.

**Methods:**

We identified duodenal T cell populations from 23 controls and 49 Rome III FD patients by flow cytometry using a surface marker antibody panel. We also analysed T cell populations in peripheral blood from 37 controls and 61 patients. Where available, we examined the number of duodenal eosinophils in patients and controls.

**Results:**

There was a shift in the duodenal T helper cell balance in FD patients compared to controls. For example, patients had increased duodenal mucosal Th2 populations in the effector (13.03 ± 16.11, 19.84 ± 15.51, *p*=0.038), central memory (23.75 ± 18.97, 37.52 ± 17.51, *p*=0.007) and effector memory (9.80±10.50 vs 20.53±14.15, *p*=0.001) populations. Th17 populations were also increased in the effector (31.74±24.73 vs 45.57±23.75, *p*=0.03) and effector memory (11.95±8.42 vs 18.44±15.63, *p*=0.027) subsets. Peripheral T cell populations were unchanged between FD and control.

**Conclusion:**

Our findings identify an association between lymphocyte populations and FD, specifically a Th2 and Th17 signature in the duodenal mucosa. The presence of effector and memory cells suggest that the microinflammation in FD is antigen driven.

## Introduction

Functional dyspepsia (FD) is a disorder of gut-brain interaction (DGBI), formerly known as functional gastrointestinal disorders (FGIDs), presenting with chronic gastrointestinal (GI) symptoms without discernible structural or biochemical abnormalities (1). In the absence of treatable pathology, FD is sub-classified into symptom groups according to the Rome criteria: post-prandial distress syndrome (PDS) and epigastric pain syndrome (EPS), with therapeutic intervention based on symptom group (2). Subtle pathologies are apparent in FD, with duodenal eosinophilia identified globally in several cohorts (3–8), and increased proportions of T cells expressing gut-homing markers (integrin α4^+^, integrin β7^+^ and chemokine receptor 9^+^, CCR9) (9), suggesting an adaptive immune involvement. Defects in duodenal mucosal barrier, measured by transepithelial electrical resistance (6) and increased permeability (6, 10, 11) are also reported. It remains unclear as to whether these defects are common to specific patient subgroups or linked to alterations in immune cell subsets.

FD patients have increased levels of CD45RA^+^CD45RO^+^ T cells (12), while the proportion of peripheral CD4^+^ lymphocytes remains unchanged (9, 12), suggesting that activation of effector cells is associated with symptoms. T cells can be sub-classified into phenotypically distinct subsets (13), and T helper (Th)2 and Th17 responses have been hypothesised as dominant in FD, given the capacity of these responses to recruit eosinophils to the duodenum (14). However to date, alterations in these subpopulations have not been shown (15). Further, no studies have demonstrated a conclusive relationship between Rome symptom subtypes and T cell subsets. FD often overlaps with the irritable bowel syndrome (IBS) (16), and while patients with multiple DGBIs have higher symptom severity scores and lower quality of life (16, 17), it is unclear whether overlap patients exhibit greater homeostatic imbalance than patients with FD only. As such, we hypothesised that a loss of GI homeostasis in FD allows for the induction of specific T cell responses that promote chronic symptoms. This study aimed to characterise T lymphocyte subsets in FD patients to identify specific immunological subsets of this condition.

## Methods

### Cohort recruitment

Participants aged 18-80 years were recruited through outpatient gastroenterology clinics at John Hunter, Gosford and Wyong Hospitals in New South Wales and Princess Alexandra Hospital, Brisbane, Queensland, Australia. All research was undertaken in accordance with approvals from the Hunter New England (reference 13/12/11/3.01) and Metro South Health (reference HREC/13/QPAH/690) Human Research Ethics Committees, with written informed consent obtained. Patients met the Rome III criteria for PDS, or EPS with or without PDS (EPS ± PDS). Given that post-prandial epigastric pain largely contributes to overlap between the Rome III subtypes (2, 18), and we had small numbers of ‘pure’ EPS subjects, we pooled these groups to create the EPS ± PDS subgroup, as previously published (19).

Asymptomatic controls required endoscopy for routine care, such as for unexplained iron deficiency anaemia (IDA), positive fecal occult blood test (+FOBT), gastroesophageal reflux disease (GERD) or dysphagia, with no organic GI disease confirmed during endoscopy. Exclusion criteria for the study included patients with a body mass index (BMI)>40, organic GI conditions and pregnant women. A medical interview captured medication, family history and demographic information. An outpatient questionnaire incorporated the Talley Bowel Disease Questionnaire (20), Nepean Dyspepsia Index (21), the Hospital Anxiety and Depression score and the Rome III questions for IBS and FD. At endoscopy, 5-7 biopsies were collected from the second portion of the duodenum (D2) and whole blood was collected in lithium heparin.

### Isolation of peripheral blood mononuclear and lamina propria mucosal cells

Density gradient centrifugation was performed to isolate peripheral blood mononuclear cells (PBMCs) as previously described (22). Within 2 hours of the endoscopic procedure and sample collection, 5 biopsies in 1x HBSS (without Ca2^+^ or Mg2^+^) were transferred into epithelial digestion buffer (1x HBSS, 5mM EDTA, 10mM HEPES) and vortexed vigorously for 5minutes. The solution was then strained through a 70uM filter and this epithelial digestion was repeated, before the supernatant containing the epithelial fraction was discarded. Biopsies were then transferred into lamina propria digestion buffer (1x HBSS, 2% FCS, 0.5mg/mL collagenase D, 10ug/mL DNAse II). The solution was vortexed vigorously for 5minutes and strained through a 70uM filter to collect the supernatant, and this digestion step was repeated. The resulting supernatant was centrifuged to pellet the mucosal immune cells (330g for 10minutes). Mucosal cells were then washed with ice-cold PBS and counted using Trypan blue. Cells were resuspended in freezing media (10% DMSO, 90% FCS; or in a 1:1 ratio of freezing media and complete RPMI-1640 media supplemented with 10% FCS, 1% HEPES, 1% L-glutamine, 1% sodium pyruvate, 0.2% penicillin-streptomycin). Cells were then frozen in a frosty-boy for at least 48 hours before transfer to liquid nitrogen storage.

### Surface marker staining for flow cytometry

After retrieval of cells from liquid nitrogen, cells were quickly thawed at 37°C until a slushie-like consistency was achieved. Cells were added dropwise to prewarmed complete RPMI-1640 media and centrifuged (330*g*, 10minutes/room temperature). Cells were then washed in PBS and centrifuged. Following removal of the supernatant, the cell pellet was resuspended in complete RPMI-1640 and transferred into a 24-well culture plate. Samples were rested at 37°C/5% CO_2_ overnight prior to staining and analysis.

Cells were resuspended at 1.0-8.0 x10^5^ cells/mL and incubated with a fixable viability dye (conjugated to AF700, 15minutes on ice), and Fc block antibody (10minutes on ice). Cells were stained (4°C/30minutes) with the antibodies (BD Biosciences, New Jersey, USA) outlined in detail in **Supplementary Table 1** and cells were fixed in 4% paraformaldehyde. Expression of surface markers was acquired on an LSRFortessa™ X20 flow cytometer with FACSDiva software (BD Biosciences) set to record 200,000 events for PBMC samples and 100,000 events for mucosal cells. Due to low mucosal cell yield and viability, if 100,000 events were unable to be obtained, a minimum of 50,000 was required for the sample to be included in analysis and analysis was only done as a percentage of the appropriate parent population to account for this. Given the lack of inflammation and low mucosal cell yields, surface marker staining was utilised over intracellular cytokine staining to improve the accuracy of lymphocyte subset identification (23, 24) and gating was performed on control samples. Data was analysed using FlowJo v.10 (BD Biosciences) and following gating of single cell and live cell populations, CD3^+^ lymphocyte populations identified on single live cells, as outlined in **Supplementary Table 2**.

### Histology

Formalin fixed, paraffin embedded (FFPE) biopsies were stained with haematoxylin and eosin (H&E) (7). Slides were digitalised using Aperio AT2 (Leica Biosystems, Wetzlar, Germany). Blinded eosinophil counts were performed by a single assessor using Aperio ImageScope (Leica Biosystems). Five high-power fields (HPFs) equivalents within a grid of 200µMx200µM, were counted in biopsies at 40x (25). Areas were selected by scanning the section for areas of increased eosinophils and counting five areas of the highest density or the single observed area of highest density and surrounding HPFs. The average number of eosinophils per HPF were calculated. Intraepithelial lymphocytes (IELs) were counted per 50 enterocytes across 3 villi and reported as the average per 50 enterocytes.

### Immunohistochemical staining

Immunohistochemical staining to assess CD117^+^ mast cells was performed by NSW Regional Biospecimen & Research Services using the Discovery Ultra Benchmark Automated Platform (Ventana Medical Systems, Inc). Cell Conditioning-1 reagent (pH 9.0, Roche, Switzerland) was used for antigen retrieval and sections were incubated for 28minutes at 37°C with anti-CD117 primary antibody (1:600, reference 4502 Aligent Dako Technologies, California, USA). Slides were incubated with an anti-rabbit secondary HQ (Roche, Switzerland) and tertiary horseradish peroxidase HQ (Roche, Switzerland). 3,3’-Diaminobenzidine (DAB) liquid substrate system (Roche, Switzerland) was used to develop sections, counterstained with haematoxylin. Slides were digitised as described, and CD117^+^ mast cells in the same manner as eosinophils, described above.

### Akoya Phenocycler multiplex immunofluorescent staining and analysis

FFPE duodenal biopsies from one representative FD patient and one control were selected (**Supplementary Table 3**) for spatial phenotyping using the Akoya Phenocycler multiplex immunofluorescence platform (26). Biopsy selection was based on increased duodenal eosinophils and proportions of mucosal cell effector Th2 and effector Th17 cells we observed in our histological and flow cytometric analysis of the broader cohort. FFPE biopsies were sectioned on Poly-L-lysine coated square glass coverslips (22x22mm) and stained with oligonucleotide-conjugated antibodies from the Akoya Biosciences pre-defined 15-marker immune core panel (CD4, CD68, CD20, CD11c, CD8, HLA-DR, CD3e, CD44, CD45, HLA-A, CD14, Ki67, Pan-CK, CD107a, CD45RO) by the Peter MacCallum Cancer Centre, Melbourne, Australia.

Slides were imaged and segmented using the Phenocycler Processor platform (Akoya Biosciences) before visualisation with QuPath (27) (version 0.3.2) and the CODEX Multiplex Analysis Viewer (MAV, version 1.5.0.8, Akoya Biosciences) plugin for Fiji (version 2.3.0/153q). Using the CODEX MAV plugin, all DAPI positive cells in the entire biopsy, antibody mean fluorescent intensity (MFI) and position (X, Y) data were imported to CytoMAP (28) in MATLAB (version R2022a update 1, MathWorks, Massachusetts, USA). Cells were then gated to determine T cell phenotypes using CD4, CD8, CD44 and CD45RO expression, and clustered into regions algorithmically by sample based on the standardised MFI of CD4, CD68, CD20, CD11c, CD8, CD3e, CD44, CD14, Pan-CK, CD107a and CD45RO (**Supplementary Table 4**) using a t-SNE algorithm.

### Statistical analysis

Datasets were analysed using Graphpad Prism 9.2 (Graphpad Software Inc., La Jolla, USA). Data was visually presented as mean ± SEM and statistics reported as mean ± SD, with *p*<0.05 considered significant. Cohort characteristics were analysed by *t-*tests. Fisher’s exact test analysed effects of confounders.

Normality was assessed using assessed by the D’Agostino-Pearson test and Grubb’s outlier test was performed on each dataset, with significant outliers excluded. Comparisons between groups were analysed by t-tests or one-way ANOVA with uncorrected Fisher’s LSD post-hoc test for normally distributed data. Non-normally distributed data was analysed by Mann-Whitney *t*-tests or Kruskal-Wallis test with Dunn’s multiple comparisons post-hoc test as appropriate.

## Results

### Cohort characteristics

For some participants, the collection of additional research biopsies was not feasible during endoscopy, or they consented to provide only a blood sample or only biopsies. In total, 48 controls and 78 FD patients were recruited for this study. Of these total participants, 12 controls (32.43% of PBMC, 52.17% of mucosal cell samples) and 32 FD patients (52.46% of PMBCs, 65.31% of mucosal cell samples) donated matched blood and biopsies for isolations of immune cells for analysis. Duodenal biopsies only were available for a further 11 controls and 17 FD patients, while 25 controls and 29 FD patients donated blood samples only for cellular isolations. This is summarized in **Supplementary Figure 1**.

#### Systemic T cell populations

PBMCs were collected from 37 controls and 61 patients (**Table 1**). Twenty patients had symptoms consistent with PDS, 6 had EPS and 35 had EPS/PDS overlap (n=41 EPS ± PDS). Twelve controls were referred to endoscopy for iron deficiency anaemia (IDA), 4 with dysphagia, 1 with reflux symptoms and 20 were undergoing faecal occult blood test (FOBT). No controls had symptoms of FD, gastrointestinal disease upon endoscopy or histological assessment. FD patients were younger than controls (56.49 ± 12.52 vs 48.57 ± 16.38, *p*=0.014) and proton pump inhibitor (PPI) usage was higher in FD (10.81% vs 69.81%, *p*<0.000). The prevalence of co-morbid Rome III IBS was significantly higher in FD (5.41% vs 45.90%, *p*<0.000).

**Table 1:** Cohort characteristics for PBMC and mucosal cell samples.

PBMC samples	Controls	FD	p value
	n=37	n=61	
Age (mean ± SD)	56.49 ± 12.52	48.57 ± 16.38	0.014*
Female (%)	19 (51.35)	42 (70.00)	0.084
BMI (mean ± SD)	27.93 ± 5.721	25.83 ± 4.425	0.068
PPI use (%)#	4 (10.81)	37 (69.81)	<0.0001****
H2RA use (%)#	0 (0.00)	5 (9.43)	0.075
NSAID use (%)#	0 (0.00)	3 (5.66)	0.266
Helicobacter pylori positive (%)#	4 (12.50)	2 (3.92)	0.200
IBS co-morbidity (%)#	2 (5.41)	28 (45.90)	<0.0001****
Mucosal cell samples	Controls	FD	p value
	n=23	n=49	
Age (mean ± SD)	52.09 ± 13.12	47.39 ± 16.52	0.199
Female (%)	11 (47.83)	37 (75.51)	0.031*
BMI (mean ± SD)	28.99 ± 5.325	25.92 ± 4.136	0.007**
PPI use (%)#	5 (23.81)	19 (45.24)	0.168
H2RA use (%)#	0 (0.00)	2 (4.76)	0.548
NSAID use (%)#	1 (4.76)	2 (4.76)	>0.9999
Helicobacter pylori positive (%)#	2 (11.11)	2 (4.88)	0.578
IBS co-morbidity (%)#	1 (4.35)	15 (30.61)	0.014*

# Denotes a factor that was not provided by all participants included in this cohort

PBMC = peripheral blood mononuclear cells, BMI = body mass index, PPI = proton pump inhibitor, H2RA = H2 receptor antagonist, NSAIDs = non-steroidal anti-inflammatory drugs

#### Duodenal biopsies

Biopsies were collected from 23 controls and 49 FD patients during upper endoscopy (**Table 1**) for mucosal cell isolations. Twenty-three patients had PDS, 6 had EPS and 20 had EPS/PDS overlap (n=26 EPS ± PDS). Indications for endoscopy among controls included IDA (n=5), dysphagia (n=3), reflux (n=5) and +FOBT (n=10). There were more females in the FD cohort (47.83% vs 75.51%, *p*=0.03) and patients had a lower BMI (28.99 ± 5.325 vs 25.92 ± 4.136, *p*=0.007). The prevalence of co-morbid IBS was higher in FD (4.35% vs 30.61%, *p*=0.014).

### FD patients have increased duodenal eosinophil counts

**Figure 1:**
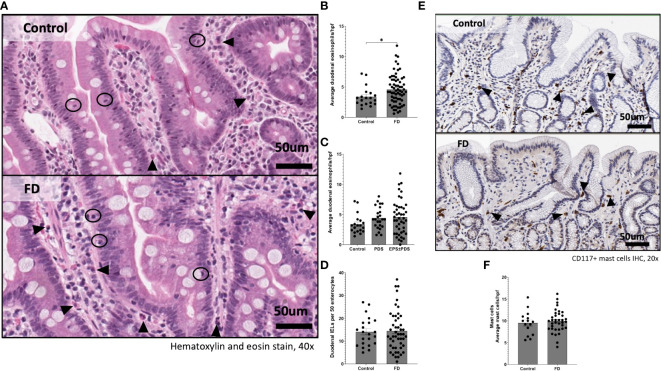
Immune hallmarks of functional dyspepsia (FD) in this cohort. Biopsies were collected from the second portion of the duodenum (D2) of FD patients and controls and stained with **(A)** haematoxylin and eosin (40x magnification, arrows indicate eosinophils, circles indicate intra-epithelial lymphocytes (IELs) scale bar=50μm). **(B)** Average duodenal eosinophil counts per 5 high powered fields were examined between controls and FD, as well as **(C)** within the FD subtypes. **(D)** Duodenal IELs were counted across groups using H&E-stained sections. **(E)** Immunohistochemical staining of CD117^+^ mast cells was also performed (20x magnification, arrows indicate CD117^+^ cells, scale bar=50μm). **(F)** Mast cells were counted based on CD117^+^ positively stained cells (brown) in all groups. n=15-22 for controls, n=38-67 for FD, n=24 PDS, n=43 EPDS ± PDS. Data presented as mean ± SEM. Statistical analysis for control vs FD, **(D, F)** parametric and **(B)** non-parametric t test, **(C)** non-parametric ANOVA. *p<0.05.

We initially aimed to examine whether our cohort exhibited previously identified pathologies for FD. Where available, histological duodenal sections for participants from which mucosal cells/PBMCs were isolated revealed no overt pathology by H&E staining (**Figure 1A**). However, duodenal eosinophils were significantly increased in biopsies from FD patients (n=19, 3.39 ± 1.61 vs n=67, 4.59 ± 2.45, *p*=0.048) compared to controls (**Figure 1B**), but unchanged between the FD subtypes (**Figure 1C**). Duodenal IELs were unchanged (**Figure 1D**) and CD117 staining for mast cells (**Figure 1E**) showed no difference between FD and controls (**Figure 1F**). These findings confirm that our cohort has the primary known histological feature of FD, duodenal eosinophilia.

### CD4^+^ effector T cells are increased in the duodenum of FD patients

**Figure 2:**
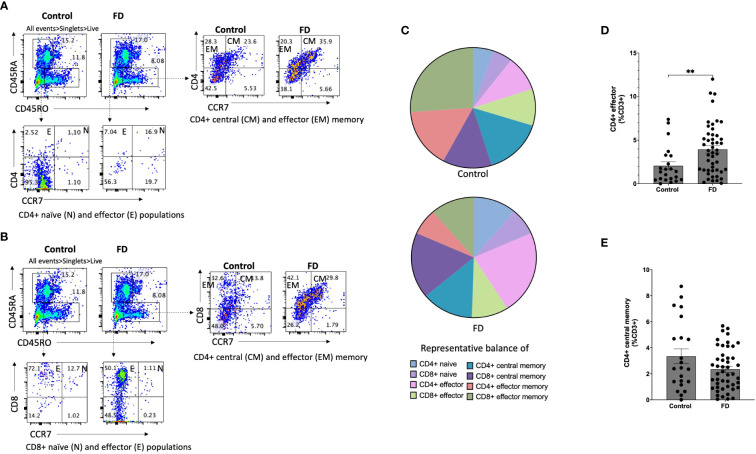
The duodenal effector and memory T cell balance in functional dyspepsia patients compared to controls. Lamina propria mucosal cells were isolated from duodenal biopsies and phenotyped using surface marker staining and flow cytometry. Naïve (CD45RA^+^ CCR7^+^), effector (CD45RA^+^ CCR7^-^), central memory (CD45RO^+^ CCR7^+^) and effector memory (CD45RO^+^ CCR7^-^) T cells were identified in the **(A)** CD4^+^ and **(B)** CD8^+^ populations. **(C)** The balance of each effector and memory population was then represented within the total control and FD cohorts before the **(D)** CD4^+^ effector and **(F)** CD4^+^ central memory populations were also investigated within these groups. n=23 controls, n=49 FD. Data presented as mean ± SEM. Statistical analysis for control vs FD, **(E)** parametric t test, **(D)** non-parametric t test. *p<0.05, **p<0.01.

Given the duodenum is implicated as a major site for symptoms in FD, we examined lymphocyte profiles in the duodenal mucosa. There were no differences in the overall proportions of CD3^+^, CD4^+^ or CD8^+^ cells between FD and controls (**Supplementary Figure 2**). We next investigated the CD4^+^ naïve, effector, central memory (CM) and effector memory (EM) subsets as a proportion of the total CD3^+^ pool based on the expression of CD45RA, CD45RO and CCR7 (**Figure 2A**) and the proportions of these subsets within the CD8^+^ population (**Figure 2B**). We identified changes in the representative balance of these cells within the mucosa (**Figure 2C**), with a statistically significant expansion of CD4^+^ effector cells in FD (2.06±2.17 vs 3.94±2.93, *p=*0.006) (**Figure 2D**). There was no change in CD4^+^CM (**Figure 2E**), CD8^+^ effector or CM cells in FD compared to controls, and the proportions of duodenal CD4^+^ or CD8^+^ naïve and EM cells were also unaffected (**Supplementary Figure 3**).

### Peripheral effector and memory T cell profiles do not reflect duodenal profiles

We used the same strategy to assess naïve, effector, CM and EM subsets in the periphery as a proportion of the CD3^+^ pool based on expression of CD45RA, CD45RO and CCR7. There were no changes the balance of PBMC effector and memory cells in FD compared to controls, however the CD8^+^ effector population was significantly decreased in FD (6.08±6.24 vs 2.85±2.55, *p*=0.027). There was also no change in the peripheral populations of CD4^+^ or CD8^+^ naïve or EM populations (**Supplementary Figure 4**). These data demonstrate that while decreased CD8^+^ effector populations are observed in FD PBMCs, increases in the CD4^+^ effector population are localised to the duodenum of FD patients.

### Populations resembling effector Th2 and Th17 cells are increased in the duodenum of FD patients

**Figure 3:**
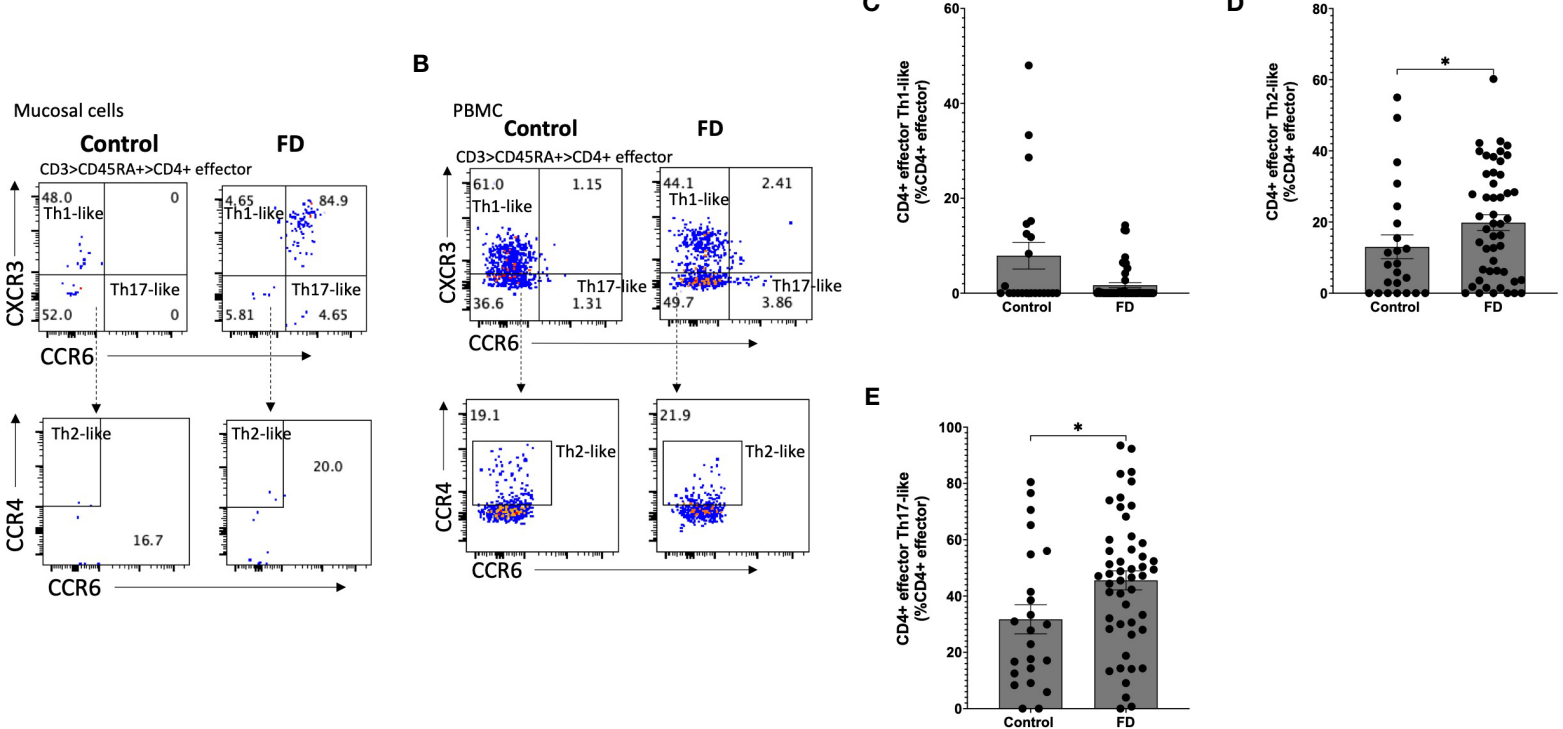
Effector T helper cells in FD patients compared to controls Lamina propria lymphocytes were isolated from duodenal biopsies and peripheral blood mononuclear cells were isolated from whole blood. Cells were phenotyped using flow cytometry. Within the CD4^+^ effector T cell (CD4^+^ CD45RA^+^ CCR7^+^) pool, T helper cell subsets were identified based on expression of CCR6, CCR4 and CXCR3 in the isolated **(A)** mucosal cells and **(B)** PBMCs. Within the duodenal effector populations, **(C)** Th1, **(D)** Th2 and **(E)** Th17 cells were investigated in FD patients compared to controls. n=23 controls, n=49 FD for mucosal cells, n=37 controls, n=61 FD for PBMCs. Data presented as mean ± SEM. Statistical analysis for control vs FD, **(E)** parametric t test, (C,D), non-parametric t test. *p<0.05.

Given the increased populations of duodenal CD4^+^ effector cells, we next characterised circulating sub-populations of Th cells as a proportion of the total effector pool. CD4^+^ effector cells were gated based on expression of CCR6, CCR4 and CXCR3 to examine the duodenal (**Figure 3A**) and peripheral (**Figure 3B**) Th subsets. There was no change in Th1-like (CCR6^-^CXCR3^+^) (**Figure 3C**), however FD patients had increased effector Th2-like (CCR6^-^CCR4^+^) cells (13.03±16.11 vs 19.84±15.51, *p*=0.038) (**Figure 3D**). In addition, effector Th17-like (CCR6^+^CCR4^+^) cells were increased in FD (31.74±24.73 vs 45.57±23.75, *p*=0.03) (**Figure 3E**). Conversely in the PBMCS, there was a decrease in the proportion of Th1-like effector cells in FD (24.13±24.16 vs 12.70±12.74, *p*=0.049) but the Th2 and Th17-like populations were unchanged (**Supplementary Figure 5**).

### Duodenal Th2 and Th17 memory T cells are also increased in FD

**Figure 4:**
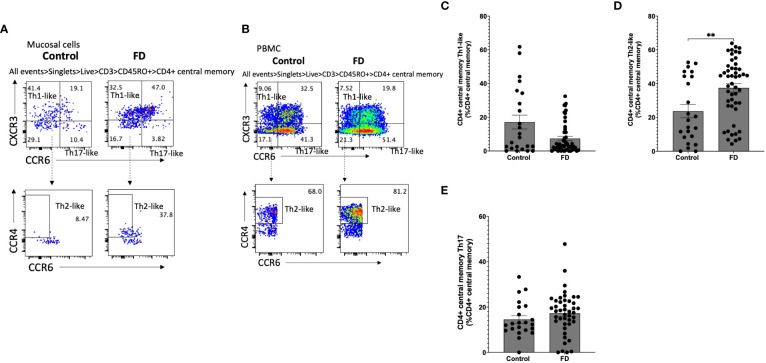
Central memory T helper cells in FD patients compared to controls Lamina propria lymphocytes were isolated from duodenal biopsies and peripheral blood mononuclear cells were isolated from whole blood. Cells were phenotyped using flow cytometry. Within the CD4^+^ central memory T cell (CD4^+^ CD45RO^+^ CCR7^+^) pool, T helper cell subsets were identified based on expression of CCR6, CCR4 and CXCR3 in the isolated **(A)** mucosal cells and **(B)** PBMCs. **(C)** Th1, **(D)** Th2 and **(E)** Th17 central memory T cells were compared within the mucosal cell central memory pool. n=23 controls, n=49 FD for LPMCS, n=37 controls, n=61 FD for PBMCs. Data presented as mean ± SEM. Statistical analysis for control vs FD, **(C,D,E)** non-parametric t test. **p<0.01.

**Figure 5:**
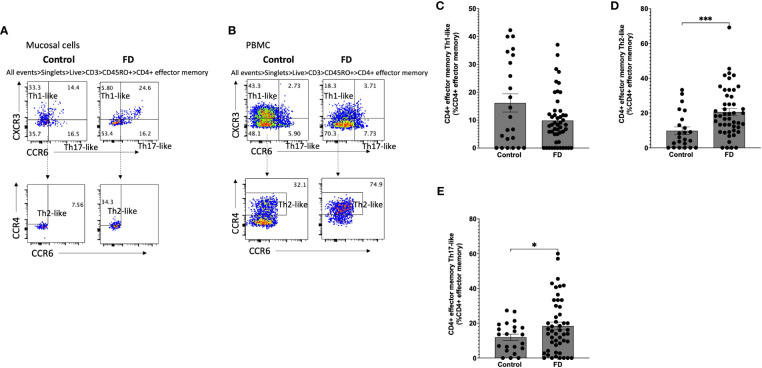
Effector memory T helper cells in FD patients compared to controls Lamina propria lymphocytes were isolated from duodenal biopsies and peripheral blood mononuclear cells were isolated from whole blood. Cells were phenotyped using flow cytometry. Within the CD4^+^ effector memory T cell (CD4^+^ CD45RO^+^ CCR7^-^) pool, T helper cell subsets were identified based on expression of CCR6, CCR4 and CXCR3 in the isolated **(A)** mucosal cells and **(B)** PBMCs. Within the mucosal cell effector memory pool, **(C)** Th1, **(D)** Th2 and **(E)** Th17 populations were investigated in FD compared to controls. n=23 controls, n=49 FD for mucosal cells, n=37 controls, n=61 FD for PBMCs. Data presented as mean ± SEM. Statistical analysis for control vs FD, **(E)** parametric t test, **(C,D)** non-parametric t test. *p<0.05, ***p<0.001.

Given we saw a duodenal Th2/Th17-like effector signature, we investigated Th subsets in the CM (CD45RO^+^CCR7^+^) pool in both mucosal cells (**Figure 4A**) and PBMCs (**Figure 4B**). Within mucosal cells, Th1-like CM populations were unchanged (**Figure 4C**), while the Th2-like CM pool was increased in FD (23.75±18.97 vs 37.52±17.51, *p*=0.007) (**Figure 4D**). There was no change in the Th17-like CM population (**Figure 4E**). We also investigated the EM (CD45RO^+^CCR7^-^) Th subpopulations in mucosal cells (**Figure 5A**) and PBMCs (**Figure 5B**). The Th1-like EM population was unchanged (**Figure 5C**) but in line with the CM population, patients had greater proportions of Th2-like EM cells (9.80±10.50 vs 20.53±14.15, *p*=0.001) (**Figure 5D**). FD mucosal cells also had increased proportions of EM Th17-like cells (11.95±8.42 vs 18.44±15.63, *p*=0.027) (**Figure 5E**).

With regards to PBMCs, FD samples had increased proportions of CM Th1-like cells (9.63±6.44 vs 15.46±11.14, *p*=0.017), while the peripheral CM Th2-like population was unchanged. The CM Th17-like (31.90±19.01 vs 20.60±18.85, *p*=0.017) population was reduced in FD. These T cell subsets were unchanged within the peripheral EM population (**Supplementary Figure 5**). Collectively, these data demonstrate the effector and memory Th1/Th2 balances are altered in FD compared to controls, with patients demonstrating a predominant Th2-like and Th17-like surface marker phenotype.

Because we saw a consistent Th2- and Th17-like phenotype in the duodenal mucosal cells in both effector and memory populations of FD patients but not in the periphery, we next compared the proportions of these subsets in the cohort of patients who had provided both blood and biopsies for cellular isolations (**Supplementary Figure 6**). While statistical power was reduced due to lower numbers, the findings supported the conclusion that signature of Th2/Th17-like populations are not consistently increased in the periphery of FD patients compared to controls, as per our total cohorts of PBMC and mucosal cell samples. As a further sensitivity analysis attempting to control for the possibility of false positives due to low event numbers in some samples, we also analysed the effector and memory T helper populations from mucosal samples where the maximum event number was recorded (n=11 controls, n=20 FD). This investigation supported our findings of a Th2- and Th17-like mucosal signature in FD (**Supplementary Figure 7**).

### Spatial distribution as an alternative method to investigate lymphocyte populations in FFPE duodenal biopsies

**Figure 6:**
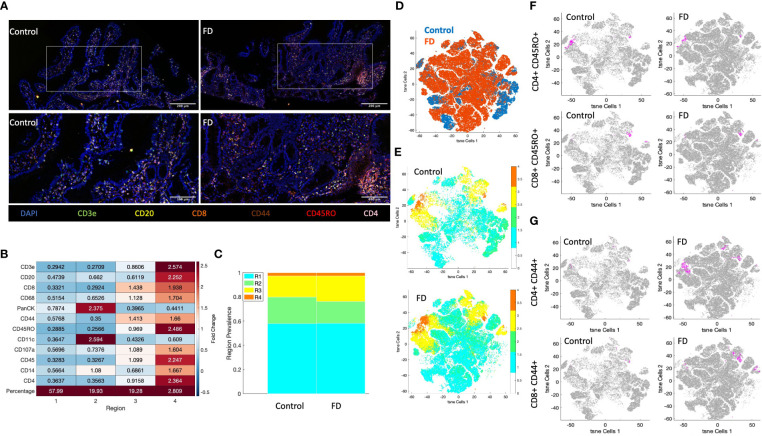
Akoya Biosciences Phenocycler investigation of lymphocytes in FD compared to control. **(A)** A duodenal biopsy from 1x control and 1x FD patient were stained using the Akoya Biosciences Phenocycler platform (top images scale bar = 200µM, bottom images = 100µM). The expression of CD3e, CD4, CD8, CD20CD44, CD45RO, and DAPI as the nuclear marker are shown. 10x and 20x magnification. **(B)** Heatmap demonstrating the fold change expression of each marker in each region generated by clustering of the DAPI positive population using CytoMAP. **(C)** The prevalence of each clustered region in the control sample compared to the FD sample. **(D)** t-SNE plot of lymphocyte-associated markers (CD3e, CD4, CD8, CD20, CD44, CD45RO) in the FD patient compared to the control. **(E)** t-SNE plot of lymphocyte-associated markers (CD3e, CD4, CD8, CD20, CD44, CD45RO) colored by clustered region in the control and FD sample. **(F)** t-SNE plots of memory lymphocytes (CD45RO^+^CD4^+^/CD8^+^) MFI intensity in control and FD sample, **(G)** t-SNE plots of activated lymphocytes (CD44^+^CD4^+^/CD8^+^) MFI intensity in control and FD sample. Magenta = positive cells, grey = negative cells. n=1 control, n=1 FD duodenal biopsy. .

In an attempt to overcome limitations of using biopsy samples for flow cytometry in microinflammatory conditions such as FD, we investigated the potential for multiplex immunochemical staining approaches to better understand spatial distribution of immune cell populations in DGBIs. FFPE duodenal biopsies from an FD patient with high effector Th2, Th17 populations and a control representative of our flow cytometry findings were stained and visualised for lymphocyte populations using CD3e, CD4, CD8, CD20, CD45RO and CD44, as well as CD68, PanCK, CD11c, CD107a and CD14. Observationally, the lymphocyte cell populations clustered together more frequently in the FD biopsy compared to the control (**Figure 6A**). All DAPI positive, marker-stained cells in the biopsies (**Supplementary Figure 8**) were clustered unsupervised by the CytoMAP software based on standardised MFI of these markers into 4 distinct regions of cells with similar spatial characteristics (**Supplementary Figure 8**) and the expression intensity of these markers in each region was analysed by heatmap (**Figure 6B**). PanCK, CD11c and CD14 were predominant in region 2, likely encompassing epithelial cells, as well as dendritic cells and/or macrophages. Interestingly, both regions 3 and 4 were dominated by immune cells, with region 4 having the highest abundance of CD3e, CD20, CD8, CD68, CD44, CD45RO, CD107a, CD45, CD14 and CD4 expression, with little PanCK or CD11c. Region 3 had increased expression of CD8 and CD44, suggesting two distinct CD8^+^ CD44^+^ populations between region 3 and 4. With regards to the prevalence of each region in the FD patient compared to control, region 2 was less abundant and region 3 was increased in the FD biopsy, while there was no difference in the prevalence of regions 1 and 4 (**Figure 6C**).

Following clustering analysis, the MFI of lymphocyte markers (CD3e, CD4, CD8, CD20, CD44 and CD45RO) were used to generate a t-SNE plot, which demonstrated differences in clustering between FD and control (**Figure 6D**). When coloured by region (**Figure 6E**), these differences are predominantly driven by region 1 and 2, while 3 and 4 clustered similarly between samples. There were no differences in the clustering patterns of memory lymphocytes (CD45RO^+^CD4^+^/CD8^+^) between FD and control (**Figure 6F**), however there were larger areas of activated cells (CD44^+^CD4^+^/CD8^+^) in FD (**Figure 6G**) similarly to our flow cytometry finding of increased in CD4^+^ effector lymphocytes in FD patients.

### Th2-like effector memory cells are increased in FD patients with IBS compared to those without

As FD is categorised based on symptom profile in the EPS and PDS subtypes, we examined the T cell subsets described above in both the periphery (**Supplementary Table 5**) and the duodenum (**Supplementary Table 6**). Interestingly, the duodenal Th2-like effector population was specifically increased in the PDS group compared to controls (13.03 ± 16.11 vs 21.22±14.58, *p*=0.04). Conversely, increased Th17-like T cells were observed in both the duodenal effector (31.74 ± 24.72 vs 49.83±25.16, *p*=0.009) and EM (11.95 ± 8.42 vs 22.03 ± 16.98, *p*=0.018) pools in EPS ± PDS compared to controls. Given the updated Rome IV criteria (released after recruitment commenced for this study) now focuses on the presence of meal related symptoms, we also analysed the effector Th2-like and Th17-like populations in patients with PDS alone and EPS with overlapping post-prandial symptoms compared to those with only EPS (**Supplementary Figure 9**). Duodenal effector Th2-like proportions were unchanged between those with and without meal-associated symptoms (18.68 ± 16.11 vs 28.12 ± 20.34, *p*=0.341). While those with meal-associated FD had increased duodenal effector Th17-like cells (31.74 ± 24.82 vs 46.28 ± 24.05, *p*=0.023) and eosinophils (3.39 ± 1.61 vs 4.71 ± 2.46, *p*=0.031) compared controls, there was no difference compared to those with EPS only.

Given the presence of multiple DGBIs is associated with greater symptom severity (16, 29), we also compared T cell profiles in the duodenum (**Supplementary Table 7**) and periphery (**Supplementary Table 8**) of FD with (FD+IBS) and without (FD-IBS) concomitant IBS. The EM Th2-like population was increased in FD+IBS compared to FD-IBS (17.85±11.50 vs 26.45±17.76, *p*=0.034), but we could not identify any further altered duodenal populations between these FD subsets, suggesting concomitant IBS and FD is associated with a greater proportion of EM Th2 cells compared to FD only.

Because there were significant differences in our cohort regarding PPI usage (**Supplementary Tables 9, 10**) and sex (**Supplementary Tables 11, 12**), we finally investigated the influence of these factors on duodenal and peripheral profiles. Interestingly, we observed that FD patients not taking PPIs had increaed duodenal EM and CM Th2-like proportions compared to controls, and that the CM Th17-like population was increased compared to FD+PPI. These findings support the notion that PPIs have immunomodulatory capacity in the duodenum however are underpowered given this was not a primary aim.

We used Spearman’s r non-parametric to investigate potential relationships between the HADS scores for anxiety and depression with duodenal T cell phenotype where data was available (n=12 controls, n=37 FD) (**Supplementary Tables 13 and 14**). Within the FD cohort but not controls, the anxiety score positively correlated with the proportion of CD8^+^ effector (r=0.41, *p*=0.03) and with the CD4^+^ effector memory Th17-like subset (r=0.37, *p*=0.02). With regards to the HADS depression score, the CD4^+^ effector memory population negatively correlated in controls (r=-0.64, *p*=0.03) but not in FD. These data suggest associations between quality of life and immune activation in the duodenum are linked and warrant further investigation.

## Discussion

Imnune activation may play a role in symptom manifestation in FD and IBS. To date no comprehensive assessment of T cell phenotypes in FD has been performed (15). As such, this study aimed to phenotype lymphocyte populations in the mucosa and periphery of FD patients to identify immune signatures of mucosal dysregulation with the intention of informing future, mechanistically focused work. Our findings have revealed a duodenal Th2 and Th17-like signature in FD compared to controls, confirming activation of specific T cell populations is a feature of the condition. Consistent with previous work and supporting the notion of FD as a disorder of homeostatic imbalance (9, 12), we did not find alterations in the proportions of total CD3^+^, CD4^+^ or CD8^+^ lymphocytes. Instead, the lymphocyte profile in FD is suggestive of subtle Th2/Th17-type responses localised to the duodenum. Interestingly, the duodenal phenotype was not observed in PBMCs, further supporting recent work demonstrating that localised responses to antigens activate the mucosal immune response in both DGBI patients and animal models of visceral hypersensitivity (30, 31). The discrepancies between the systemic and mucosal response warrant caution when interpreting immune profiles from unstimulated PBMCs in these conditions.

Given Th2 immune responses are associated with eosinophil recruitment and activation (32), our findings suggest a specific antigen response is linked to duodenal eosinophil increases in FD. We also identified increased proportions of Th17-like cells in the duodenum, a subset traditionally associated with autoimmune conditions, including coeliac disease (33) and rheumatoid arthritis (34); or the adaptive immune response to extracellular pathogens (35). The concept of multiple Th subsets activated in the mucosa has a precedence in asthma, where inflammatory signatures have been implicated in simultaneous activation of Th17 and Th2 responses (36). An existing theory is that these overlapping responses are the result of multiple environmental antigen exposures, such as a concurrent infection and exposure to allergen; or that these responses represent generation of autoimmune reactions due to chronic cycles of inflammation and repair (37). This scenario is plausible in FD, given that FD can develop after acute gastroenteritis, and is also associated with atopic and autoimmune conditions (38–40). Unfortunately, we did not have data on the proportion of our cohort with a post-infectious (PI) onset of FD to compare the T cell population with non-PI FD, however this is an important question that remains to be answered. Another hypothesis from asthma suggests Th17 cells are activated where Th2 responses are ineffective at clearing an infection or antigen (36). Th17 cells then regulate Th2 cells to restore homeostasis (41), but failure to resolve the response may drive cyclic symptoms. As such, we would propose a multi-antigen model for immune activation in FD. In this hypothetical scenario, disruption to duodenal homeostasis (such as infection) would drive physiological disruption in the gut and allow for greater contact between mucosal immune cells and luminal antigens. Once the stimulus is removed and the immune response lapses, Th2 and Th17 memory cells remain in the duodenum for future exposures.

While some of our cohort had overlapping IBS, these patients were recruited to the study only where their FD symptoms were the primary complaint. Our data suggests effector memory cells with a Th2-like phenotype are more abundant in FD-IBS overlap, compared to FD only, however additional studies where this comparison is the primary aim are required to elucidate the importance of this difference. Importantly, this difference may link to known increased symptom severity in patients with more than one DGBI (16, 29). While we identified significant differences in cell populations between FD and controls, our cohort is underpowered to effectively examine these cell populations under the Rome criteria subtypes. We had small numbers of ‘pure’ EPS subjects, as most reported both EPS and PDS symptoms, and it is recognised that post-prandial epigastric pain largely contributes to this overlap in the Rome III criteria (2, 18). Additional analyses on those with ‘pure’ EPS compared to those with meal-associated symptoms (as per the suggestions of Rome IV), demonstrated no difference in the proportion of effector Th2- and Th17-like cells. This suggests that grouping of patient symptoms does not align well with biological differences, however this requires confirmation in cohorts appropriately powered for subtype analysis. One remaining question is whether the Th2 and Th17-like signatures reported in the duodenum are associated with reported co-morbid atopy or autoimmune conditions, given the associations between these conditions and DGBIs (38, 42). However, data on these conditions was unavailable for a large subset of our study cohort, meaning we were unable to perform characterisation or association analyses on these features to investigate potential links further.

There are limitations to our study, including the smaller number of controls and their outpatient status. The ideal phenotyping study would compare to healthy controls, however there are ethical and logistical considerations in obtaining duodenal biopsies from healthy individuals. However, we believe careful characterisation of both our controls and FD cohort for organic diseases and immune confounders (including BMI and age) has mitigated this limitation. Of note, this study used Rome III for consistency, as recruitment commenced prior to Rome IV but the updated criteria are similar (43). It should be noted that variations in tissue structures in biopsies, as well as the selection of one patient and one control for multiplex immunofluorescent staining limits the conclusion that can be drawn from this experiment, and so should be validated with a larger number of samples. However, to our knowledge, this is the first application of such technologies to investigate the spatial microenvironment in DGBIs, which we have shown warrants further study to understand the complex immunopathologies that underlie these common conditions. In addition, limitations in the amount of biopsy material and the absence of frank inflammation in the tissue prevented lymphocyte phenotyping *via* transcription factor and intracellular cytokine staining. Ideally, future targeted studies would use this approach and investigate regulatory T cells in FD, given these exist in balance with Th17 cells (44). Further, limited cell numbers prevented the use of fluorescence minus one controls for gating. However, given the overall paucity of lymphocyte profiling in the DGBI field, we believe these findings are important, novel observations that provide confirmation that Th2 and Th17-like responses are implicated in FD pathogenesis. We believe the broad overview of T cell populations examined here provides the framework for more focused characterisation studies moving forward.

This study demonstrates that FD patients have increased proportions of specific effector T cells in the duodenum. Further, for the first time, we have shown patients have increased Th2 and Th17-like cells, confined to the duodenum. Importantly neither Rome criteria subgrouping nor IBS overlap account for this immunophenotype in our cohort. Our findings suggest dual lymphocyte response pathways are involved in FD symptom generation, giving new insights into the aetiology of this condition.

## Data availability statement

The original contributions presented in the study are included in the article/**Supplementary Material**. Further inquiries can be directed to the corresponding author.

## Ethics statement

The studies involving human participants were reviewed and approved by Hunter New England (reference 13/12/11/3.01) Metro South Health (reference HREC/13/QPAH/690). The patients/participants provided their written informed consent to participate in this study.

## Author contributions

SK, NT, MW and GH participated in the design of the concept, hypothesis, and aims of the study. GB and SK participated in initial drafting of the manuscript. GB performed sample processing, immunophenotyping experiments, cytokine assays and analysis. JB, AM, KM, and TF assisted with collection and processing of patient samples. GB, RC and MW performed histological analysis. PN advised on data analysis. MP, SB, MI, LG, RF, MV, AS, and NT assisted with recruitment and review of cohort. PF and JH assisted with resources, experimental design and manuscript editing. CN, NP, NT, MW assisted with concept, experimental design and manuscript editing. All the authors read and approved the final manuscript. All authors contributed to the article and approved the submitted version.
