# Supplementary material for: Type 2 and type 17 effector cells are increased in the duodenal mucosa but not peripheral blood of patients with functional dyspepsia
Source: Front Immunol. 2023 Jan 6;13:1051632. doi: 10.3389/fimmu.2022.1051632 (PMC9852875; doi:10.3389/fimmu.2022.1051632)
Supplement: Supplementary file 1 [file DataSheet_1.pdf]

Supplementary Table 1: List of antibodies used in surface marker flow cytometry staining for identification of lymphocyte subsets

Antibody target	Fluorophore	Clone	Supplier	Vol./100µL test
CD3	BUV805	UCHT1	BD Biosciences, cat no. 612896	2µL
CD4	FITC	RPA-T4	BD Biosciences, cat no. 555346	5µL
CD8	BUV496	RPA-T8	BD Biosciences, cat no. 612942	2µL
CD45RA	BUV395	5H9	BD Biosciences, cat no. 740315	2µL
CD45RO	PE-CY7	UCHL1	BD Biosciences, cat no. 560608	2µL
CCR7 (CD197)	BV711	150503	BD Biosciences, cat no. 566602	5µL
CCR6 (CD196)	BV786	11A9	BD Biosciences, cat no. 563704	5µL
CCR4 (CD194)	BV421	1G1	BD Biosciences, cat no. 562579	5µL
CXCR3 (CD183)	PE	1C6	BD Biosciences, cat no. 557185	15µL
Integrin α4	PE-CF594	9F10	BD Biosciences, cat no. 563645	5µL
Integrin β7	BV650	FIB504	BD Biosciences, cat no. 564285	5µL
CCR9	APC	112509	BD Biosciences, cat no. 557975	2µL
Fixable viability dye	FVS700	N/A	BD Biosciences, cat no. 564997	1:1000

Supplementary Table 2: Surface markers for the identification of T cell subsets

T cell subset	Surface maker phenotype
Lymphocytes	$CD3^+$
T helper cells	$CD3^+CD4^+$
Cytotoxic T cells	$CD3^+CD8^+$
Naïve T cells	$CD4^+CD45RA^+CCR7^+/CD8^+CD45RA^+CCR7^+$
Effector T cells	$CD4^+CD45RA^+CCR7^-/CD8^+CD45RA^+CCR7^-$
Central memory T cells	$CD4^+CD45RO^+CCR7^+/CD8^+CD45RO^+$ $CCR7^+$
Effector memory T cells	$CD4^+CD45RO^+CCR7^-/CD8^+CD45RO^+CCR7^-$
Small intestinal homing T cells	$CD4^+\alpha4^+\beta7^+CCR9^+/CD8^+\alpha4^+\beta7^+CCR9^+$
Effector Th1-like T cells	$CD4^+CD45RA^+CCR7^-CCR6^-CXCR3^+$
Effector Th2-like T cells	$CD4^+CD45RA^+CCR7^-CCR6^-CCR4^+$
Effector Th17-like T cells	$CD4^+CD45RA^+CCR7^-CCR6^+CCR4^+$
Effector gut-homing T cells	$CD4^+CD45RA^+CCR7^-\alpha4^+\beta7^+CCR9^+/CD8^+CD45RA^+CCR7^-\alpha4^+\beta7^+CCR9^+$
Central memory Th1-like T cells	$CD4^+CD45RO^+CCR7^+CCR6^-CXCR3^+$
Central memory Th2-like T cells	$CD4^+CD45RO^+CCR7^+CCR6^-CCR4^+$
Central memory Th17-like T cells	$CD4^+CD45RO^+CCR7^+CCR6^+CCR4^+$
Central memory gut-homing T cells	$CD4^+CD45RO^+CCR7^+\alpha4^+\beta7^+CCR9^+/CD8^+CD45RO^+CCR7^+\alpha4^+\beta7^+CCR9^+$
Effector memory Th1-like T cells	$CD4^+CD45RO^+CCR7^-CCR6^-CXCR3^+$
Effector memory Th2-like T cells	$CD4^+CD45RO^+CCR7^-CCR6^-CCR4^+$
Effector memory Th17-like T cells	$CD4^+CD45RO^+CCR7^-CCR6^+CCR4^+$
Effector memory gut-homing T cells	$CD4^+CD45RO^+CCR7^-\alpha4^+\beta7^+CCR9^+/CD8^+CD45RO^+CCR7^-\alpha4^+\beta7^+CCR9^+$

Supplementary Table 3: Characteristics of duodenal biopsies selected for Phenocycler analysis

	**Control**	**FD**
n	1	1
Age	63	50
Sex	M	M
Eosinophils/hpf*	11	34
Effector mucosal cells (% $CD3^+$)	1.68	1.36
Effector Th2 mucosal cells (% effector mucosal cells) *	0.00	1.81
Effector Th17 mucosal cells (% effector mucosal cells) *	17.10	26.34
Peripheral $CD4^+$ gut-homing T cells (% total $CD4^+$)	0	0.03
PPI usage	No	No
Co-morbid IBS?	No	No
H. pylori status	Negative	Negative
Self-reported wheat sensitive?	No	No

* = factors primarily considered in sample selection

Supplementary Table 4: Akoya Biosciences Phenocycler targets

Marker	Populations identified
DAPI	Nuclear stain
CD3e	Lymphocytes
CD20	B cells
CD8	Cytotoxic T cells
CD68	Macrophages
PanCK	Epithelial cells
CD44	Activated T cells
CD45RO	Memory T cells
CD11c	Dendritic cells
CD107a	NK cells, $CD8^+$ T cells
CD14	Macrophages/monocytes
CD4	T helper cells

Supplementary Table 5: Peripheral T cell populations in FD patients and controls analysed by FD subtype

	**Control (mean±SD)**	**PDS (mean±SD)**	**EPS±PDS (mean±SD)**	**Control vs. PDS (*p* value)**	**Control vs. EPS±PDS (*p* value)**	**PDS vs. EPS±PDS (*p* value)**
	n=37	n=20	n=41			
CD3+	41.13±13.70	50.00±23.28	46.90±19.04	0.098	0.189	0.553
CD4+	45.63±18.70	44.29±17.83	43.44±15.49	0.783	0.566	0.857
CD8+	23.21±15.15	17.03±16.65	22.14±13.80	0.246	0.97	0.258
CD4+ effector	1.59±2.07	2.06±2.52	1.30±1.36	0.461	0.913	0.399
CD4+ effector Th1	24.13±24.16	8.29±6.97	15.23±15.42	0.027*	0.183	0.247
CD4+ effector Th2	16.20±12.34	19.66±11.42	17.21±15.75	0.176	0.774	0.107
CD4+ effector Th17	4.93±6.59	7.72±9.76	4.88±6.42	0.526	0.856	0.62
CD4+ naïve	15.35±12.94	15.76±15.61	18.50±12.92	0.821	0.277	0.257
CD8+ effector	6.08±6.24	2.35±3.46	3.10±3.61	0.035*	0.081	0.493
CD8+ naïve	5.06±4.24	5.16±5.66	9.23±8.56	0.824	0.041*	0.058
CD4+ central memory	9.40±6.68	10.37±10.68	12.84±9.46	0.707	0.107	0.33
CD4+ central memory Th1	9.63±6.44	17.84±13.11	14.30±10.01	0.023*	0.056	0.468
CD4+ central memory Th2	11.79±7.12	13.15±11.54	14.69±11.29	0.867	0.524	0.481
CD4+ central memory Th17	31.90±19.01	18.32±20.99	21.71±17.89	0.035*	0.048*	0.618
CD4+ effector memory	4.13±4.06	2.36±2.90	3.59±3.80	0.132	0.658	0.243
CD4+ effector memory Th1	27.84±18.22	21.43±14.35	25.77±16.67	0.147	0.677	0.259
CD4+ effector memory Th2	12.71±10.86	9.53±9.37	8.51±9.43	0.123	0.232	0.569

CD4+ effector memory Th17	4.70±3.87	3.67±4.81	4.10±4.46	0.295	0.445	0.659
CD8+ central memory	2.20±2.08	1.34±1.63	2.71±2.22	0.085	0.257	0.007**
CD8+ effector memory	1.99±2.48	1.27±2.17	1.64±1.60	0.089	0.911	0.102

Supplementary Table 6: Duodenal T cell populations in FD patients and controls analysed by FD subtype

	**Control (mean±SD)**	**PDS (mean±SD)**	**EPS±PDS (mean±SD)**	**Control vs. PDS (*p* value)**	**Control vs. EPS±PDS (*p* value)**	**PDS vs. EPS±PDS (*p* value)**
	n=23	n=23	n=26			
CD3+	39.90±10.69	37.28±12.76	46.19±14.89	0.616	0.08	0.024*
CD4+	32.57±11.12	27.97±16.73	29.56±10.92	0.269	0.458	0.706
CD8+	25.67±16.05	18.07±12.70	18.96±8.97	0.044*	0.071	0.817
CD4+ effector	2.06±2.17	3.13±2.34	5.07±3.71	0.116	0.001**	0.093
CD4+ effector Th1	7.90±13.22	1.69±3.72	0.77±2.06	0.368	0.088	0.424
CD4+ effector Th2	13.03±16.11	21.22±14.58	18.61±16.48	0.04*	0.113	0.599
CD4+ effector Th17	31.74±24.72	40.76±21.590	49.83±25.16	0.203	0.009**	0.187
CD4+ naïve	1.18±1.08	2.14±2.50	1.19±2.44	0.137	0.11	0.962
CD8+ effector	2.15±3.95	0.53±0.34	1.67±4.70	0.000**	0.192	0.165
CD8+ naïve	1.12±1.00	1.15±0.96	1.33±1.10	0.688	0.3	0.542
CD4+ central memory	3.35±2.68	1.93±1.78	2.68±1.40	0.013	0.216	0.167
CD4+ central memory Th1	17.14±19.76	9.93±10.79	5.04±6.93	0.473	0.032	0.158
CD4+ central memory Th2	23.75±18.97	35.86±19.37	38.98±16.00	0.042*	0.01*	0.621
CD4+ central memory Th17	14.54±7.76	13.97±8.49	17.73±7.69	0.815	0.178	0.113
CD4+ effector memory	3.49±4.91	1.34±1.89	1.13±1.31	0.241	0.229	0.986
CD4+ effector memory Th1	16.14±15.65	9.70±8.58	10.0±9.88	0.399	0.355	0.956
CD4+ effector memory Th2	9.80±10.50	22.73±16.52	18.68±11.81	0.002**	0.009**	0.483
CD4+ effector memory Th17	11.95±8.42	14.39±13.15	22.03±16.98	0.573	0.018*	0.067

CD8+ central memory	2.78±1.59	2.33±2.18	3.72±2.15	0.478	0.127	0.025*
CD8+ effector memory	5.70±8.79	1.66±4.18	1.56±2.93	0.421	0.347	0.914

Supplementary Table 7: Duodenal T cell populations in FD patients with and without concomitant IBS (%)

	Control (mean±SD)	FD IBS^{-ve} (mean±SD)	FD IBS^{+ve} (mean±SD)	Control vs. FD IBS^{-ve} (*p* value)	Control vs. FD IBS^{+ve} (*p* value)	FD IBS^{-ve} vs. FD IBS^{+ve} (*p* value)
	n=23	n=34	n=15			
CD3+	39.30±10.69	40.56±14.20	45.29±15.14	0.729	0.183	0.259
CD4+	32.57±11.12	28.41±14.89	29.73±11.45	0.261	0.543	0.769
CD8+	25.67±16.05	18.81±11.42	17.83±9.64	0.064	0.096	0.826
CD4+ effector	2.06±2.17	3.68±2.76	4.52±3.28	0.024*	0.010*	0.437
CD4+ effector Th1-like	7.90±13.22	1.87±4.15	1.28±2.62	0.165	0.251	0.994
CD4+ effector Th2-like	13.03±16.11	19.03±16.80	21.67±12.42	0.098	0.035*	0.417
CD4+ effector Th17-like	31.74±24.72	46.33±24.69	43.84±22.19	0.029*	0.137	0.741
CD4+ naïve	1.18±1.08	1.63±1.43	2.29±2.51	0.201	0.076	0.425
CD8+ effector	2.15±3.95	1.30±4.19	0.79±0.61	0.026*	0.204	0.523
CD8+ naïve	1.11±1.00	1.16±0.99	1.20±0.67	0.689	0.291	0.434
CD4+ central memory	3.35±2.68	2.37±1.66	2.229±1.55	0.083	0.132	0.909
CD4+ central memory Th1-like	17.14±19.76	8.12±10.13	5.76±6.85	0.162	0.119	0.659
CD4+ central memory Th2-like	23.75±18.97	37.20±17.73	38.23±17.72	0.014*	0.038*	0.941
CD4+ central memory Th17-like	14.54±7.76	17.81±9.96	14.04±7.40	0.182	0.867	0.174
CD4+ effector memory	3.49±4.91	1.09±1.47	1.20±1.35	0.134	0.384	0.724
CD4+ effector memory Th1-like	16.14±15.65	10.41±8.96	6.58±6.30	0.510	0.100	0.232
CD4+ effector memory Th2-like	9.80±10.50	17.85±11.50	26.45±17.76	0.023*	0.000***	0.034*
CD4+ effector memory Th17-like	11.95±8.41	18.52±15.62	18.28±16.21	0.090	0.180	0.956

CD8+ central memory	2.78±1.59	3.10±2.38	3.03±2.00	0.577	0.718	0.916
CD8+ effector memory	5.70±8.79	2.17±4.74	0.70±0.68	0.449	0.282	0.618

Supplementary Table 8: Peripheral T cell populations in FD patients with and without concomitant IBS (%)

	Control (mean±SD)	FD IBS^{-ve} (mean±SD)	FD IBS^{+ve} (mean±SD)	Control vs. FD IBS^{-ve} (*p* value)	Control vs. FD IBS^{+ve} (*p* value)	FD IBS^{-ve} vs. FD IBS^{+ve} (*p* value)
	n=37	n=33	n=28			
CD3+	41.13±13.70	48.44±26.06	47.31±10.87	0.101	0.184	0.811
CD4+	45.63±18.70	40.51±17.89	47.23±13.26	0.220	0.708	0.133
CD8+	23.21±15.15	18.25±14.53	22.99±14.96	0.311	0.880	0.283
CD4+ effector	1.59±2.07	1.28±1.73	1.62±1.49	0.466	0.298	0.091
CD4+ effector Th1-like	24.13±24.16	9.71±12.10	17.85±14.84	0.007**	0.774	0.026*
CD4+ effector Th2-like	16.20±12.34	20.35±16.80	14.11±8.57	0.401	0.637	0.217
CD4+ effector Th17-like	4.93±6.59	9.14±10.90	2.97±2.83	0.229	0.688	0.131
CD4+ naïve	15.35±12.94	17.26±13.90	18.00±13.90	0.557	0.438	0.834
CD8+ effector	6.07±6.24	2.10±3.25	3.28±3.00	0.003**	0.400	0.048*
CD8+ naïve	5.06±4.24	7.95±7.40	7.34±7.80	0.161	0.319	0.731
CD4+ central memory	9.40±6.68	10.48±9.91	13.78±9.68	0.613	0.050	0.149
CD4+ central memory Th1-like	9.63±6.44	15.17±11.12	15.80±11.36	0.057	0.029*	0.721
CD4+ central memory Th2-like	11.79±7.12	16.35±13.34	11.64±7.78	0.349	0.731	0.224
CD4+ central memory Th17-like	31.90±19.01	16.70±17.09	25.19±20.08	0.005**	0.244	0.146
CD4+ effector memory	4.13±4.06	2.03±2.57	4.11±3.65	0.043*	0.872	0.035*
CD4+ effector memory Th1-like	27.84±18.22	19.25±15.26	30.35±14.85	0.031*	0.541	0.009**
CD4+ effector memory Th2-like	12.71±10.86	8.05±9.85	11.23±8.65	0.041*	0.774	0.104
CD4+ effector memory Th17-like	4.70±3.87	2.87±3.83	5.22±5.01	0.062	0.807	0.047*

CD8+ central memory	2.20±2.08	2.29±2.31	2.46±2.17	0.995	0.679	0.700
CD8+ effector memory	1.67±1.54	0.963±1.23	1.99±1.99	0.108	0.678	0.055

Supplementary Table 9: Duodenal T cell populations in FD patients and controls taking proton pump inhibitors (%)

	**Control (mean±SD)**	**FD PPI^{-ve} (mean±SD)**	**FD PPI^{+ve} (mean±SD)**	**Control vs. FD PPI^{-ve} (*p* value)**	**Control vs. FD PPI^{+ve} (*p* value)**	**FD PPI^{-ve} vs. FD PPI^{+ve} (*p* value)**
	n=24	n=21	n=19			
CD3+	38.90±10.64	43.16±14.64	43.74±13.41	0.273	0.226	0.888
CD4+	32.46±10.88	27.84±13.09	29.87±16.38	0.281	0.536	0.657
CD8+	25.83±15.72	17.01±10.21	20.39±12.49	0.042*	0.200	0.458
CD4+ effector	1.99±2.15	4.17±3.08	4.19±3.12	0.011*	0.013	0.973
CD4+ effector Th1-like	7.90±13.22	0.87±2.15	2.54±4.42	0.043*	0.769	0.098
CD4+ effector Th2-like	13.08±15.75	24.78±16.68	16.58±14.15	0.010*	0.300	0.159
CD4+ effector Th17-like	31.02±24.43	44.33±21.84	48.98±27.27	0.074	0.020*	0.551
CD4+ naïve	1.15±1.07	1.75±1.31	2.68±2.82	0.091	0.29*	0.589
CD8+ effector	2.20±3.93	0.63±0.44	0.67±0.55	0.018*	0.028*	0.920
CD8+ naïve	1.10±0.98	1.23±0.89	1.47±1.30	0.479	0.251	0.650
CD4+ central memory	3.31±2.62	2.18±1.66	3.41±3.80	0.182	0.898	0.259
CD4+ central memory Th1-like	17.35±19.35	4.63±5.31	11.29±13.68	0.039	0.464	0.211
CD4+ central memory Th2-like	23.19±18.75	39.73±17.62	34.96±19.32	0.008**	0.068	0.465
CD4+ central memory Th17-like	14.97±7.86	19.94±8.49	14.03±9.32	0.050	0.905	0.047*
CD4+ effector memory	3.39±4.82	0.59±0.48	1.71±2.05	0.011*	0.796	0.034*
CD4+ effector memory Th1-like	16.61±15.47	8.94±9.35	7.75±5.52	0.161	0.294	0.965
CD4+ effector memory Th2-like	9.77±10.27	19.90±15.06	22.98±14.44	0.014*	0.001**	0.395
CD4+ effector memory Th17-like	13.36±10.65	23.22±18.25	13.73±12.08	0.080	0.997	0.095

CD8+ central memory	2.80±1.55	2.92±2.58	3.42±2.07	0.850	0.352	0.462
CD8+ effector memory	6.28±9.06	0.48±0.57	2.39±4.66	0.032*	0.730	0.100

Supplementary Table 10: Peripheral T cell populations in FD patients and controls taking proton pump inhibitors (%)

	**Control (mean±SD)**	**FD PPI^{-ve} (mean±SD)**	**FD PPI^{+ve} (mean±SD)**	**Control vs. FD PPI^{-ve} (*p* value)**	**Control vs. FD PPI^{+ve} (*p* value)**	**FD PPI^{-ve} vs. FD PPI^{+ve} (*p* value)**
	n=37	n=37	n=16			
CD3+	41.13±13.70	48.64±18.36	49.90±26.42	0.194	0.023*	0.205
CD4+	45.63±18.70	46.16±16.19	39.24±11.72	0.891	0.215	0.181
CD8+	23.21±15.15	23.18±14.85	19.00±14.82	0.789	0.506	0.388
CD4+ effector	1.59±2.07	1.47±1.72	1.16±1.17	0.843	0.603	0.501
CD4+ effector Th1-like	24.13±24.16	14.11±11.65	10.40±14.13	0.248	0.026*	0.190
CD4+ effector Th2-like	16.20±12.34	17.93±14.38	17.92±13.60	0.766	0.553	0.720
CD4+ effector Th17-like	4.93±6.59	3.76±4.45	12.42±13.00	0.792	0.060	0.037*
CD4+ naïve	15.35±12.94	19.60±13.46	17.61±15.88	0.186	0.584	0.628
CD8+ effector	6.08±6.24	3.31±3.76	2.54±3.76	0.129	0.035*	0.359
CD8+ naïve	5.06±4.24	9.15±7.95	6.40±7.21	0.041*	0.745	0.220
CD4+ central memory	9.40±6.68	12.16±8.60	16.51±14.81	0.196	0.123	0.592
CD4+ central memory Th1-like	9.63±6.44	16.78±11.78	11.02±6.36	0.001**	0.619	0.041*
CD4+ central memory Th2-like	11.79±7.12	11.42±6.90	15.88±15.24	0.860	0.135	0.103
CD4+ central memory Th17-like	31.90±19.01	24.67±19.70	13.47±16.29	0.195	0.005**	0.073
CD4+ effector memory	4.13±4.06	3.72±3.52	1.66±2.11	0.957	0.036*	0.039*
CD4+ effector memory Th1-like	27.84±18.22	28.29±14.23	15.59±15.31	0.906	0.013*	0.010*
CD4+ effector memory Th2-like	12.71±10.86	10.93±8.04	9.29±12.82	0.457	0.266	0.596
CD4+ effector memory Th17-like	4.70±3.87	5.01±4.74	2.12±3.69	0.870	0.026*	0.018*

CD8+ central memory	2.20±2.08	2.42±2.11	2.98±2.60	0.693	0.303	0.469
CD8+ effector memory	1.67±1.54	1.73±1.88	0.88±1.05	0.901	0.193	0.225

Supplementary Table 11: Duodenal T cell populations in FD patients and controls analysed by sex (%)

	**Control (Female) (mean±SD)**	**Control (Male) (mean±SD)**	**FD (Female) (mean±SD)**	**FD (Male) (mean±SD)**	**Control female vs. Control male (*p* value)**	**Control female vs. FD female (*p* value)**	**Control female vs. FD male (*p* value)**	**Control male vs. FD female (*p* value)**	**Control male vs. FD male (*p* value)**	**FD female vs. FD male (*p* value)**
	n=11	n=12	n=37	n=11						
CD3+	44.99±9.14	36.24±6.25	42.91±15.45	39.04±11.73	0.120	0.643	0.287	0.140	0.616	0.390
CD4+	30.85±11.15	34.13±111.34	29.97±13.98	25.74±13.54	0.548	0.848	0.360	0.354	0.127	0.361
CD8+	22.76±15.63	28.34±16.64	19.25±12.41	16.55±3.99	0.572	0.407	0.406	0.122	0.158	0.861
CD4+ effector	2.52±2.46	1.20±0.99	4.27±3.57	3.73±2.21	0.225	0.152	0.223	0.002**	0.013*	0.949
CD4+ effector Th1-like	4.31±10.51	7.52±9.67	1.69±3.72	0.70±1.48	0.293	0.834	0.731	0.121	0.161	0.825
CD4+ effector Th2-like	11.66±9.95	14.28±20.62	21.53±15.47	14.03±15.68	0.692	0.073	0.726	0.172	0.969	0.171
CD4+ effector Th17-like	44.07±28.25	20.44±14.32	45.15±24.39	46.21±23.63	0.019*	0.894	0.832	0.002**	0.011*	0.896
CD4+ naïve	1.57±1.30	0.63±0.35	1.92±2.02	2.07±2.37	0.074	0.777	0.742	0.0126*	0.038*	0.900
CD8+ effector	0.96±0.84	4.89±7.41	1.29±4.02	0.73±0.38	0.143	0.293	0.908	0.004**	0.114	0.363
CD8+ naïve	1.49±1.32	0.75±0.36	1.49±2.06	1.07±0.62	0.180	0.780	0.820	0.162	0.282	0.992
CD4+ central memory	2.40±2.01	4.14±2.98	2.62±2.96	2.60±1.50	0.172	0.987	0.591	0.083	0.402	0.506
CD4+ central memory Th1-like	47.10±7.41	25.93±21.89	8.15±9.71	3.25±1.95	0.004**	0.344	0.663	0.006**	0.014*	0.689
CD4+ central memory Th2-like	27.20±20.82	20.59±17.39	36.92±18.32	38.90±16.14	0.390	0.126	0.138	0.009**	0.019*	0.753
CD4+ central memory Th17-like	15.83±8.99	11.66±2.79	16.56±10.20	17.73±3.54	0.266	0.812	0.619	0.099	0.107	0.701
CD4+ effector memory	0.78±1.02	4.77±5.14	1.08±1.33	1.13±0.79	0.004**	0.383	0.209	0.006**	0.117	0.486

CD4+ effector memory Th1-like	12.37±14.90	19.60±16.15	9.88±10.42	8.91±2.08	0.197	0.900	0.662	0.080	0.417	0.512
CD4+ effector memory Th2-like	7.15±8.33	12.23±11.99	20.10±15.98	20.80±5.47	0.300	0.005**	0.002**	0.115	0.035*	0.301
CD4+ effector memory Th17-like	10.84±10.75	13.06±5.52	18.18±15.46	19.11±17.62	0.516	0.165	0.244	0.562	0.607	0.954
CD8+ central memory	2.57±1.77	2.95±1.47	2.97±2.38	3.08±1.57	0.666	0.587	0.573	0.978	0.883	0.880
CD8+ effector memory	0.98±1.80	7.63±8.66	2.25±4.96	1.29±1.35	0.013*	0.294	0.139	0.039*	0.319	0.430

Supplementary Table 12: Peripheral T cell populations in FD patients and controls analysed by sex (%)

	Control (Female) (mean±SD)	Control (Male) (mean±SD)	FD (Female) (mean±SD)	FD (Male) (mean±SD)	Control female vs. Control male (*p* value)	Control female vs. FD female (*p* value)	Control female vs. FD male (*p* value)	Control male vs. FD female (*p* value)	Control male vs. FD male (*p* value)	FD female vs. FD male (*p* value)
	n=19	n=18	n=42	n=19						
CD3+	42.83±12.02	39.44±15.36	48.83±20.89	45.90±19.64	0.887	0.198	0.436	0.146	0.356	0.701
CD4+	42.87±20.56	48.54±16.60	44.11±16.89	42.76±14.50	0.319	0.795	0.985	0.365	0.317	0.782
CD8+	17.96±12.36	28.76±16.15	17.35±13.81	28.08±14.71	0.033*	0.932	0.035*	0.018*	0.970	0.020*
CD4+ effector	2.17±2.84	1.04±0.96	1.531±1.53	1.22±1.85	0.420	0.961	0.347	0.375	0.907	0.396
CD4+ effector Th1-like	26.44±27.82	18.19±13.97	12.18±11.68	16.23±18.01	0.930	0.193	0.409	0.176	0.373	0.740
CD4+ effector Th2-like	12.80±9.32	17.82±12.66	17.96±13.25	15.11±10.86	0.254	0.190	0.753	0.975	0.409	0.347
CD4+ effector Th17-like	4.95±7.32	3.71±3.225	5.33±8.11	8.60±10.02	0.580	0.708	0.088	0.779	0.263	0.102
CD4+ naïve	14.44±13.40	16.32±12.75	19.72±14.92	11.32±6.92	0.663	0.148	0.470	0.358	0.254	0.025*
CD8+ effector	4.14±4.44	7.19±6.68	2.29±2.82	4.15±4.67	0.162	0.255	0.749	0.005**	0.280	0.129
CD8+ naïve	4.71±4.68	5.44±3.82	7.40±7.23	8.68±8.27	0.393	0.167	0.095	0.716	0.427	0.568
CD4+ central memory	9.35±6.96	9.47±6.55	13.18±11.56	11.32±8.91	0.960	0.390	0.470	0.375	0.451	0.992
CD4+ central memory Th1-like	9.79±7.43	9.46±5.50	17.44±11.78	9.656±5.52	0.914	0.004**	0.964	0.003**	0.964	0.003**
CD4+ central memory Th2-like	12.88±8.68	10.57±4.82	15.76±12.08	10.70±8.65	0.544	0.636	0.306	0.246	0.700	0.094
CD4+ central memory Th17-like	30.26±19.23	33.63±19.17	16.46±17.09	29.75±19.78	0.580	0.008**	0.932	0.001**	0.524	0.011*
CD4+ effector memory	3.22±4.18	5.05±3.83	2.45±2.69	4.81±4.65	0.102	0.760	0.218	0.025*	0.671	0.076
CD4+ effector memory Th1-like	28.83±19.00	26.80±17.85	24.81±15.77	23.33±16.75	0.717	0.394	0.321	0.679	0.536	0.753

CD4+ effector memory Th2-like	11.72±10.95	13.76±10.98	9.31±8.98	10.05±10.46	0.537	0.389	0.615	0.119	0.270	0.795
CD4+ effector memory Th17-like	3.90±3.86	5.59±3.81	3.37±3.37	5.35±5.56	0.204	0.686	0.509	0.062	0.541	0.243
CD8+ central memory	1.89±1.84	2.07±1.46	2.49±2.44	2.11±1.72	0.607	0.512	0.581	0.970	0.982	0.991
CD8+ effector memory	1.00±0.89	2.21±1.73	1.06±1.34	2.24±2.05	0.063	0.941	0.057	0.024*	0.993	0.019*

Supplementary Table 13: Spearman's r correlations between HADS anxiety score and duodenal mucosal lymphocyte populations

	Control (n=12)			FD (n=37)		
	**r**	**95% confidence interval**	***p* value**	**r**	**95% confidence interval**	***p* value**
CD4	0.1168	-0.5045 to 0.6584	0.7163	0.1829	-0.1874 to 0.5078	0.3163
CD8	-0.1274	-0.6645 to 0.4964	0.6911	0.1896	-0.1808 to 0.5129	0.2987
CD4 effector	-0.01062	-0.5936 to 0.5797	0.9779	0.1554	-0.1872 to 0.4643	0.3584
CD4 effector Th1	Horizontal line			0.03577	-0.3007 to 0.3643	0.8335
CD4 effector Th2	-0.4752	-0.8304 to 0.1546	0.1194	-0.07616	-0.3989 to 0.2634	0.6541
CD4 effector Th17	0.1876	-0.4485 to 0.6975	0.5558	-0.1487	-0.4589 to 0.1938	0.3797
CD4 naive	-0.2797	-0.7443 to 0.3673	0.3749	0.1994	-0.1430 to 0.4992	0.2367
CD8 effector	-0.4567	-0.8229 to 0.1776	0.1367	0.4063	0.08487 to 0.6511	0.0126*
CD8 naive	-0.2443	-0.7268 to 0.3997	0.4403	0.1755	-0.1672 to 0.4803	0.2989
CD4 central memory	0.06217	-0.5444 to 0.6260	0.8468	0.2191	-0.1227 to 0.5145	0.1926
CD4 central memory Th1	-0.191	-0.6993 to 0.4457	0.5455	0.1461	-0.1964 to 0.4567	0.3883
CD4 central memory Th2	-0.1735	-0.6900 to 0.4601	0.5865	-0.07478	-0.3978 to 0.2647	0.66
CD4 central memory Th17	-0.2726	-0.7408 to 0.3739	0.3876	-0.02725	-0.3569 to 0.3084	0.8728
CD4 effector memory	-0.4	-0.7992 to 0.2439	0.1968	0.0316	-0.3045 to 0.3607	0.8527
CD4 effector memory Th1	-0.1946	-0.7012 to 0.4427	0.5396	-0.272	-0.5547 to 0.06692	0.1034
CD4 effector memory Th2	-0.4991	-0.8399 to 0.1239	0.1009	0.3164	-0.01844 to 0.5874	0.0564
CD4 effector memory Th17	-0.3812	-0.7910 to 0.2647	0.2187	0.3716	0.04422 to 0.6270	0.0235*
CD8 central memory	0.03369	-0.5642 to 0.6084	0.9177	0.2056	-0.1366 to 0.5040	0.2222
CD8 effector memory	-0.1826	-0.6949 to 0.4526	0.5645	-0.04361	-0.3711 to 0.2935	0.7977

Supplementary Table14: Spearman's r correlations between HADS depression score and duodenal mucosal lymphocyte populations

	Control (n=12)			FD (n=37)		
	**r**	**95% confidence interval**	**P (two-tailed)**	**r**	**95% confidence interval**	**P (two-tailed)**
CD4	0.06714	-0.5409 to 0.6291	0.8366	0.1521	-0.2179 to 0.4838	0.4061
CD8	0.09895	-0.5178 to 0.6480	0.7593	0.1791	-0.1913 to 0.5048	0.3268
CD4 effector	0.04947	-0.5533 to 0.6182	0.8807	0.1394	-0.2029 to 0.4514	0.4104
CD4 effector Th1	Horizontal line			-0.0873	-0.4083 to 0.2529	0.6074
CD4 effector Th2	-0.6337	-0.8896 to -0.07472	0.0305*	-0.0904	-0.4109 to 0.2500	0.5947
CD4 effector Th17	0.2686	-0.3777 to 0.7389	0.395	-0.07657	-0.3993 to 0.2630	0.6524
CD4 naive	-0.1307	-0.6663 to 0.4938	0.6837	0.1795	-0.1631 to 0.4835	0.2877
CD8 effector	-0.2226	-0.7158 to 0.4188	0.4828	0.2029	-0.1394 to 0.5019	0.2285
CD8 naive	-0.06361	-0.6269 to 0.5434	0.8456	0.181	-0.1616 to 0.4847	0.2836
CD4 central memory	-0.06915	-0.6303 to 0.5394	0.8297	0.2031	-0.1392 to 0.5021	0.2279
CD4 central memory Th1	-0.3471	-0.7758 to 0.3009	0.2644	0.1164	-0.2252 to 0.4325	0.4927
CD4 central memory Th2	-0.1166	-0.6583 to 0.5046	0.7168	-0.05079	-0.3773 to 0.2869	0.7653
CD4 central memory Th17	-0.08834	-0.6418 to 0.5256	0.7848	0.09736	-0.2434 to 0.4167	0.5665
CD4 effector memory	-0.6431	-0.8929 to -0.09064	0.0278*	0.02037	-0.3146 to 0.3509	0.9047
CD4 effector memory Th1	-0.3628	-0.7829 to 0.2845	0.2438	0.0628	-0.2758 to 0.3876	0.7119
CD4 effector memory Th2	-0.3559	-0.7798 to 0.2918	0.2535	0.2095	-0.1326 to 0.5070	0.2134
CD4 effector memory Th17	-0.2212	-0.7151 to 0.4200	0.4836	0.2793	-0.05904 to 0.5601	0.0941
CD8 central memory	-0.06018	-0.6248 to 0.5458	0.8518	0.2187	-0.1231 to 0.5142	0.1934
CD8 effector memory	-0.2814	-0.7451 to 0.3657	0.3701	0.0053	-0.3282 to 0.3376	0.9752

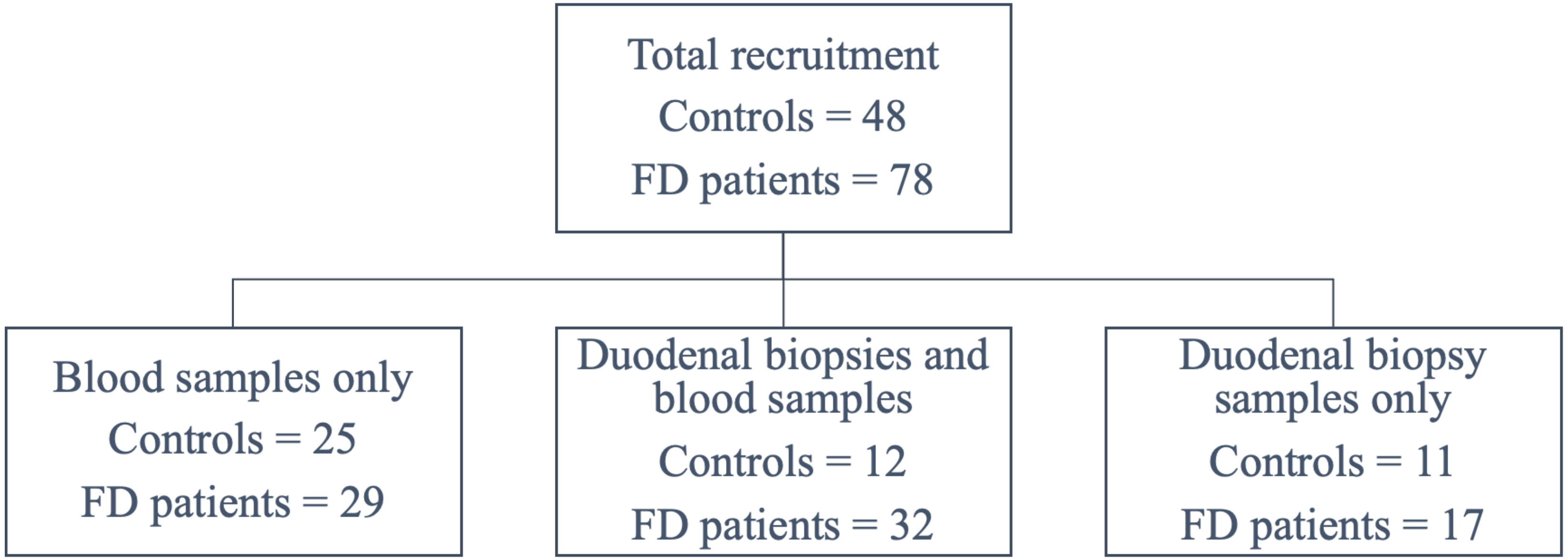
Total recruitment
Controls = 48
FD patients = 78
Blood samples only
Controls = 25
FD patients = 29
Duodenal biopsies and blood samples
Controls = 12
FD patients = 32
Duodenal biopsy samples only
Controls = 11
FD patients = 17

Supplementary Figure 1: Flow chart for recruitment and collection of samples from FD patients and outpatient controls.

In total, 48 controls and 78 FD patients were recruited for this study. Within this cohort, there were a total of 37 control and 61 FD patient PBMC samples; and 23 control and 49 FD LPMC samples available for analysis. Of the total participants recruited 12 controls (32.43% of total PBMCs, 52.17% of total LPMCs) and 32 FD patients (52.46% of total PMBCs, 65.31% of total LPMCs) donated both blood and biopsies for isolations of immune cells for analysis, while duodenal biopsies only were available for 11 controls and 17 FD patients. 25 controls and 29 FD patients donated blood samples only, without biopsies for cellular isolations.

Supplementary Figure 2

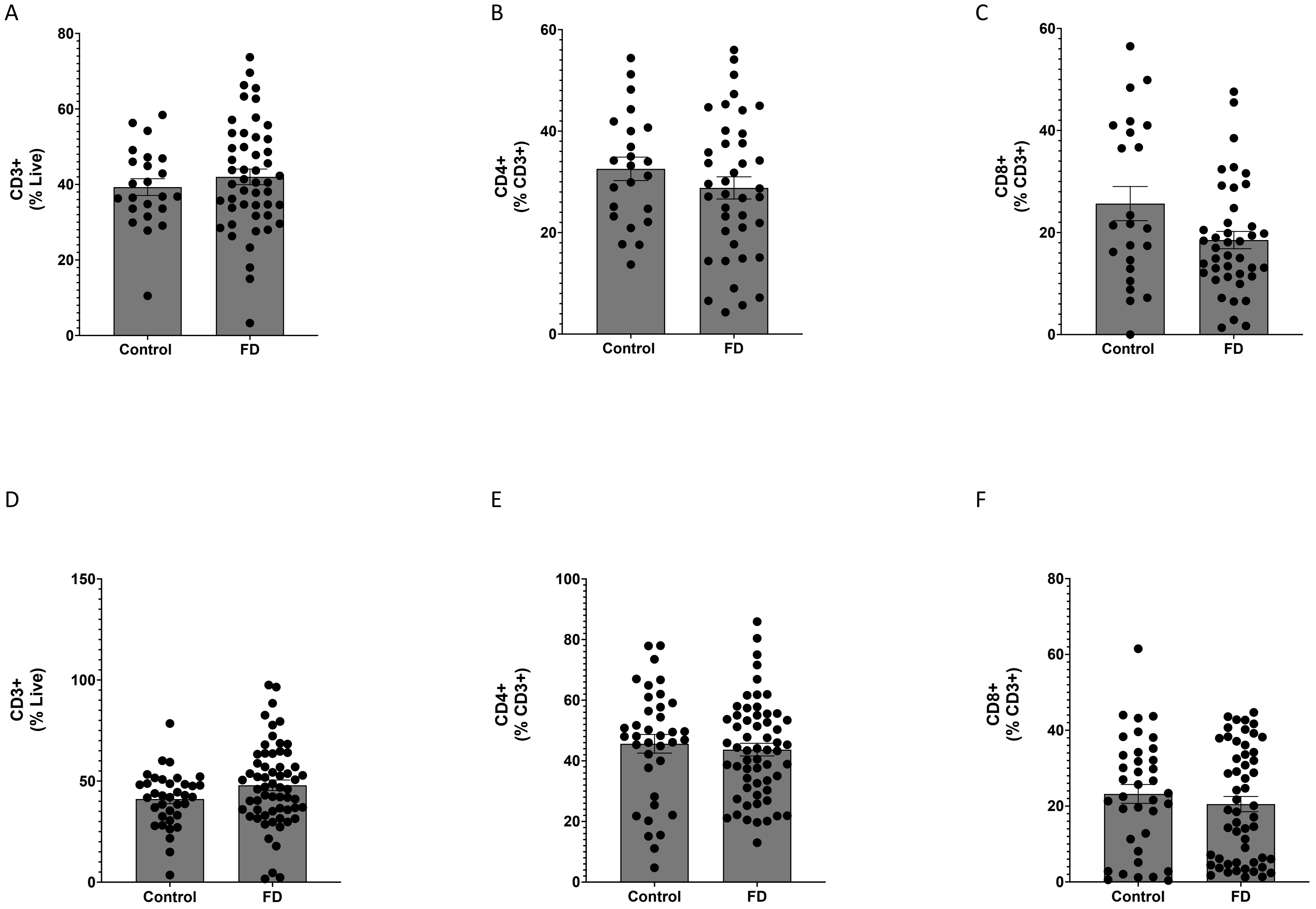

Supplementary Figure 2: Duodenal and peripheral lymphocytes in functional dyspepsia.

Lamina propria lymphocytes were isolated from duodenal biopsies and peripheral blood mononuclear cells were isolated from whole blood. Cells were phenotyped using flow cytometry. In the mucosal cells, (A) $CD3^+$, (B) $CD4^+$ and (C) $CD8^+$ populations were investigated, along with the PBMC (D) $CD3^+$, (E) $CD4^+$ and (F) $CD8^+$ populations in FD patients compared to controls. n=23 controls, n=49 FD for mucosal cells, n=37 controls, n=61 FD for PBMCs. Data presented as mean±SEM. Statistical analysis for control vs FD, (A,B,C,D,E) parametric t test, (F) non-parametric t test. *p<0.05.

Supplementary Figure 3

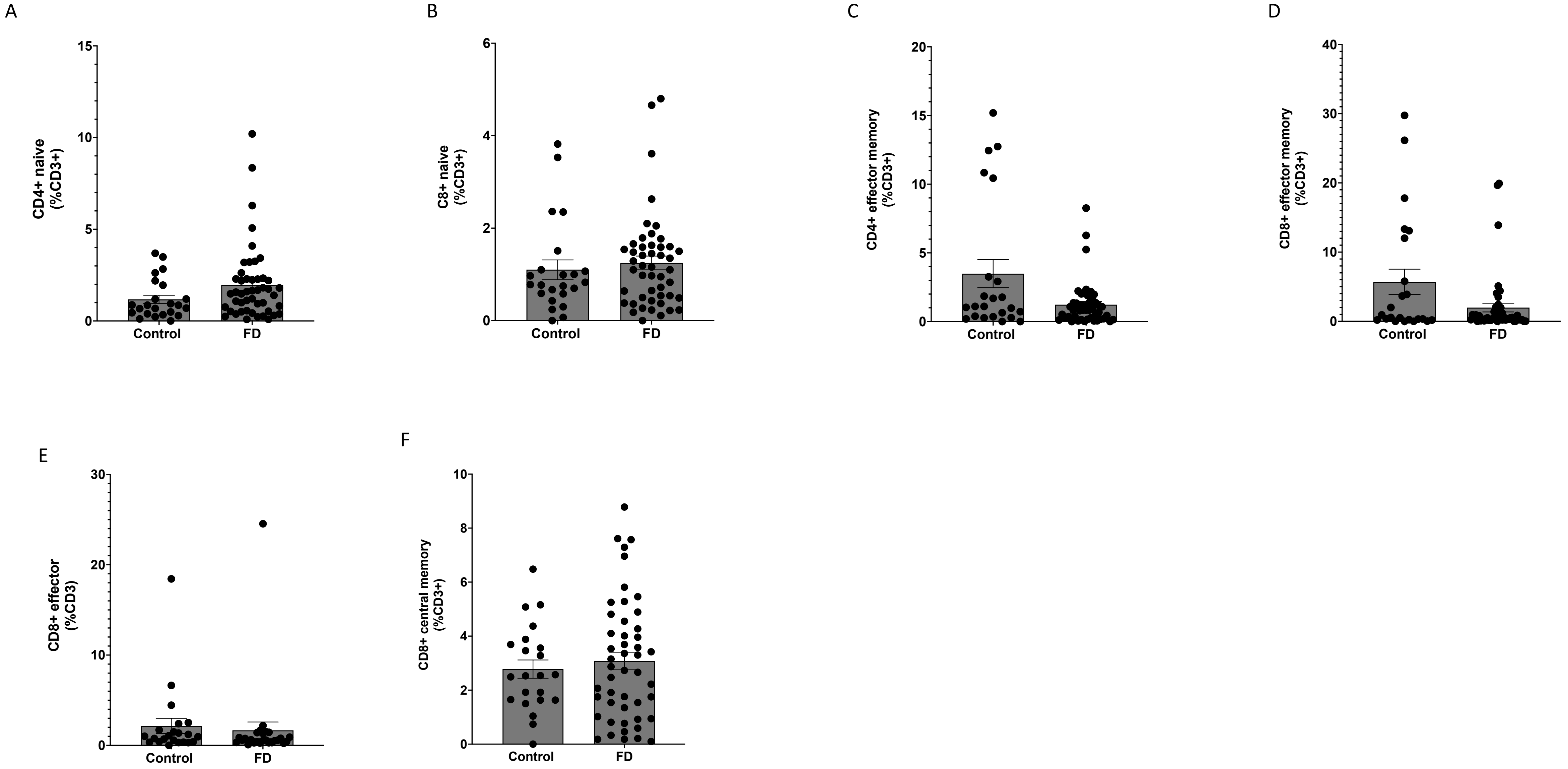

Supplementary Figure 3: Duodenal and peripheral naïve and effector memory T cell populations in FD patients compared to controls.

Lamina propria mononuclear cells were isolated from duodenal biopsies and phenotyped using surface marker staining and flow cytometry. (A) $CD4^+$ and (B) $CD8^+$ naïve ($CD45RA^+$ $CCR7^+$), along with (C) $CD4^+$ and (D) $CD8^+$ effector memory ($CD45RO^+$ $CCR7^-$) T cell populations were compared between controls and FD patients. The (E) $CD8^+$ effector and (F) $CD8^+$ central memory populations were also investigated within these groups. n=23 controls, n=49 FD. Data presented as mean±SEM. Statistical analysis for control vs FD, (F) parametric *t* test, (A,B,C,D,E) non-parametric t test.

Supplementary Figure 4

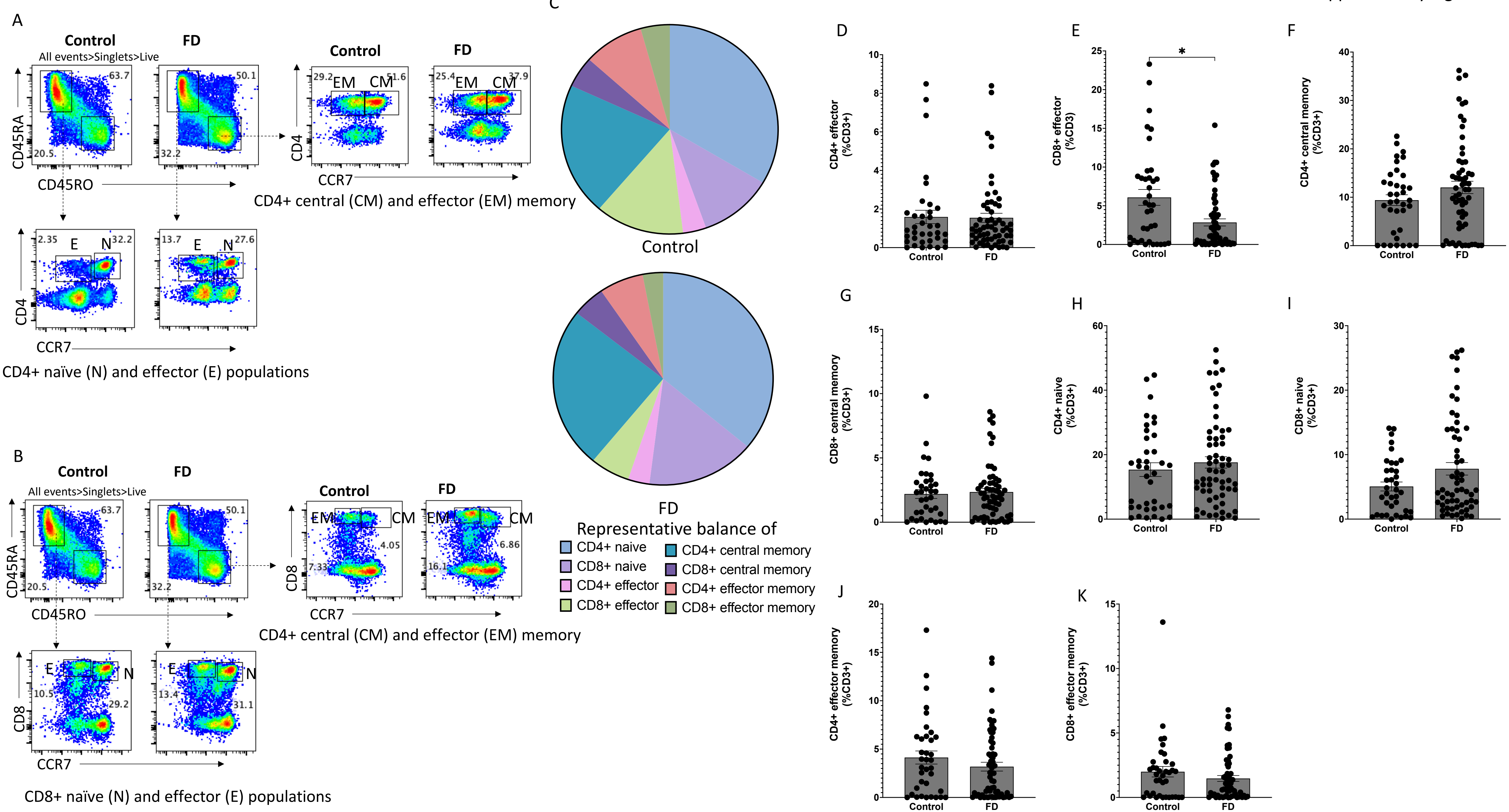

Supplementary Figure 4: The peripheral effector and memory T cell balance in FD compared to controls.

Peripheral blood mononuclear cells were isolated using density gradient centrifugation and phenotyped using surface marker staining and flow cytometry. Peripheral naïve ($CD45RA^+$ $CCR7^+$), effector ($CD45RA^+$ $CCR7^-$), central memory ($CD45RO^+$ $CCR7^+$) and effector memory ($CD45RO^+$ $CCR7^-$) T cells were identified in the (A) $CD4^+$ and (B) $CD8^+$ populations. (C) The balance of each effector and memory population was then represented within the control and FD cohorts before the (D) $CD4^+$ and (E) $CD8^+$ effector populations were compared between controls and FD. The (F) $CD4^+$ central memory and (G) $CD8^+$ central memory populations were also investigated within these groups, in addition to the (H) $CD4^+$ and (I) $CD8^+$ naïve ($CD45RA^+$ $CCR7^+$), (J) $CD4^+$ and (K) $CD8^+$ effector memory ($CD45RO^+$ $CCR7^-$) T cell populations in control and FD PBMCs.

n=37 controls, n=61 FD. Data presented as mean±SEM. Statistical analysis for control vs FD, (F) parametric t test, (D,E,G,H,I,J,K) non-parametric t test. *p<0.05, **p<0.01.

Supplementary Figure 5

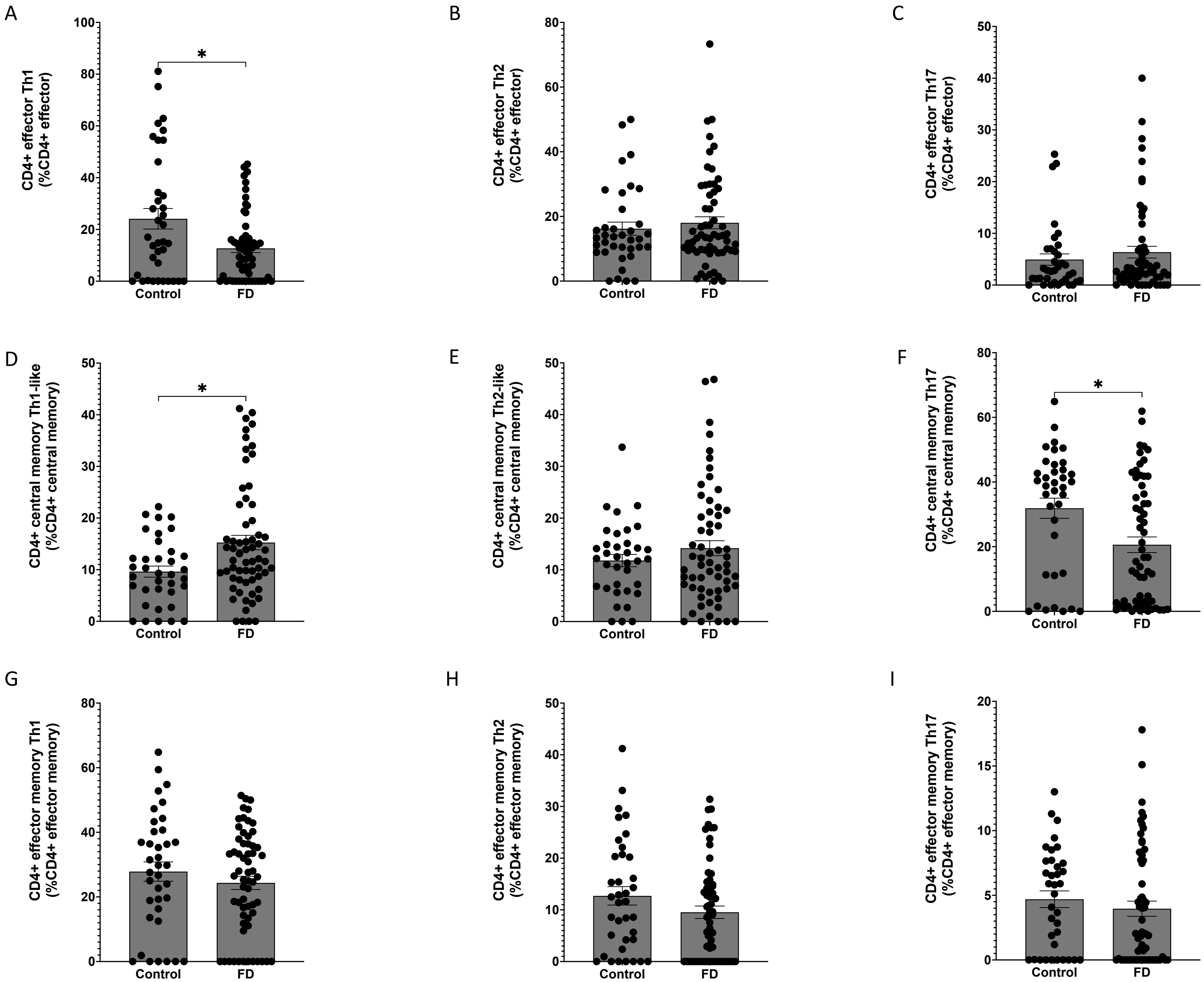

Supplementary Figure 5: T helper subsets in FD patients compared to controls.

Peripheral blood mononuclear cells were isolated from whole blood. Cells were phenotyped using flow cytometry. Within the $CD4^+$ effector T cell ($CD45RA^+$ $CCR7^+$), central memory ($CD45RO^+$ $CCR7^+$) and effector memory $CD45RO^+$ $CCR7^-$) pools, T helper cell subsets were identified based on expression of CCR6, CCR4 and CXCR3. Within the peripheral effector populations, (A) Th1-like, (B) Th2-like and (C) Th17-like cells were investigated in FD patients compared to controls. (D) Th1-like, (E) Th2-like and (F) Th17-like central memory, as well as (G) Th1-like, (H) Th2-like and (I) Th17-like effector memory T cell populations were also investigated in this cohort. n=37 controls, n=61 FD for PBMCs. Data presented as mean±SEM. Statistical analysis for control vs FD, non-parametric t test.*$p<0.05$.

Supplementary Figure 6

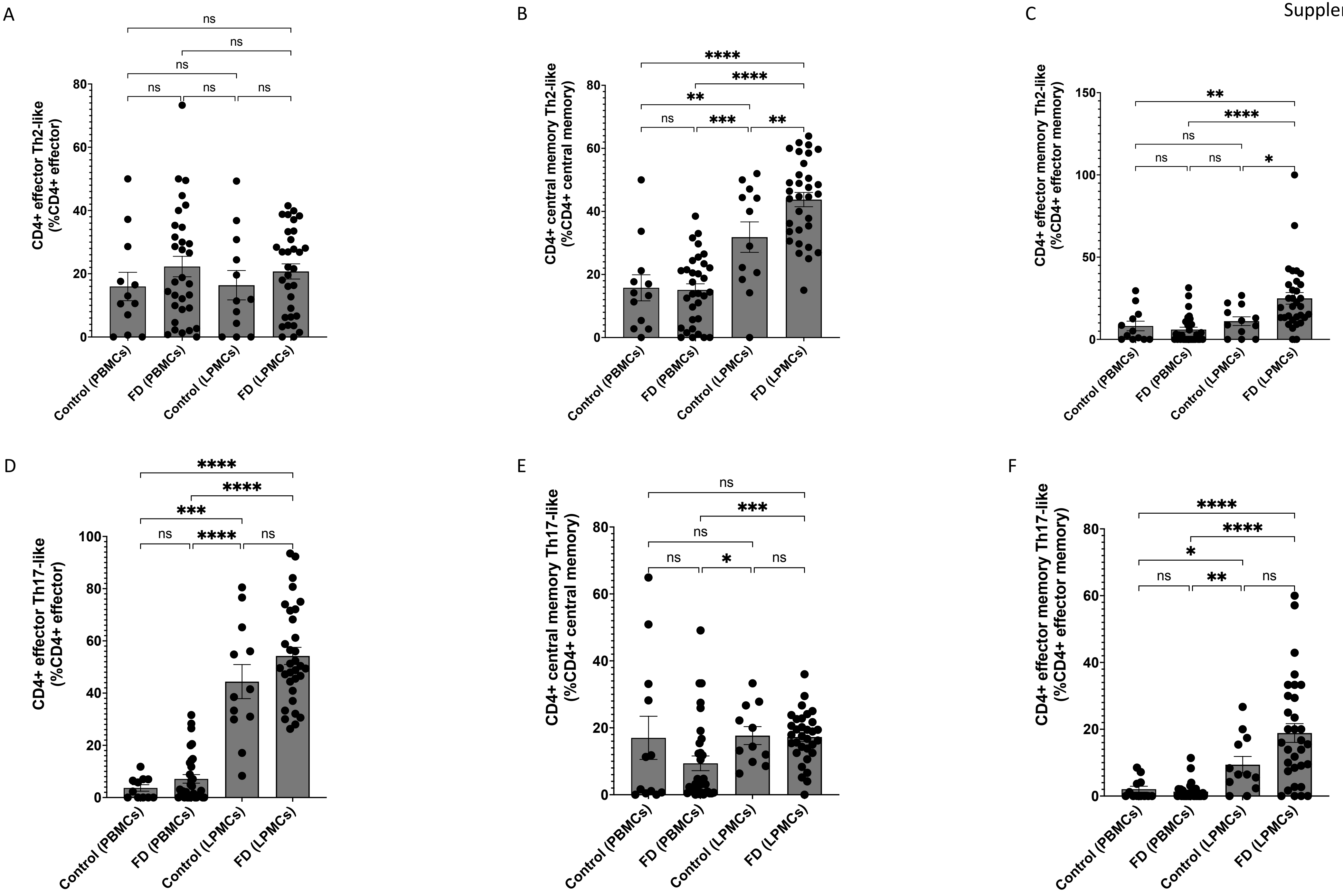

Supplementary Figure 6: Comparison of control and FD peripheral blood and duodenal mucosal cell populations.

Analysis of participants who provided matched blood and biopsy samples for analysis by flow cytometry were investigated for $CD4^+$ Th2-like cells in the (A) effector, (B) central memory and (C) effector memory populations; as well as the $CD4^+$ Th17-like populations in the (D) effector, (E) central memory and (F) effector memory pools. n=12 controls, n=32 for FD. Data presented as mean±SEM. Statistical analysis: Non-parametric ANOVA. *p<0.05, **p<0.01, ***p<0.001.

Supplementary Figure 7

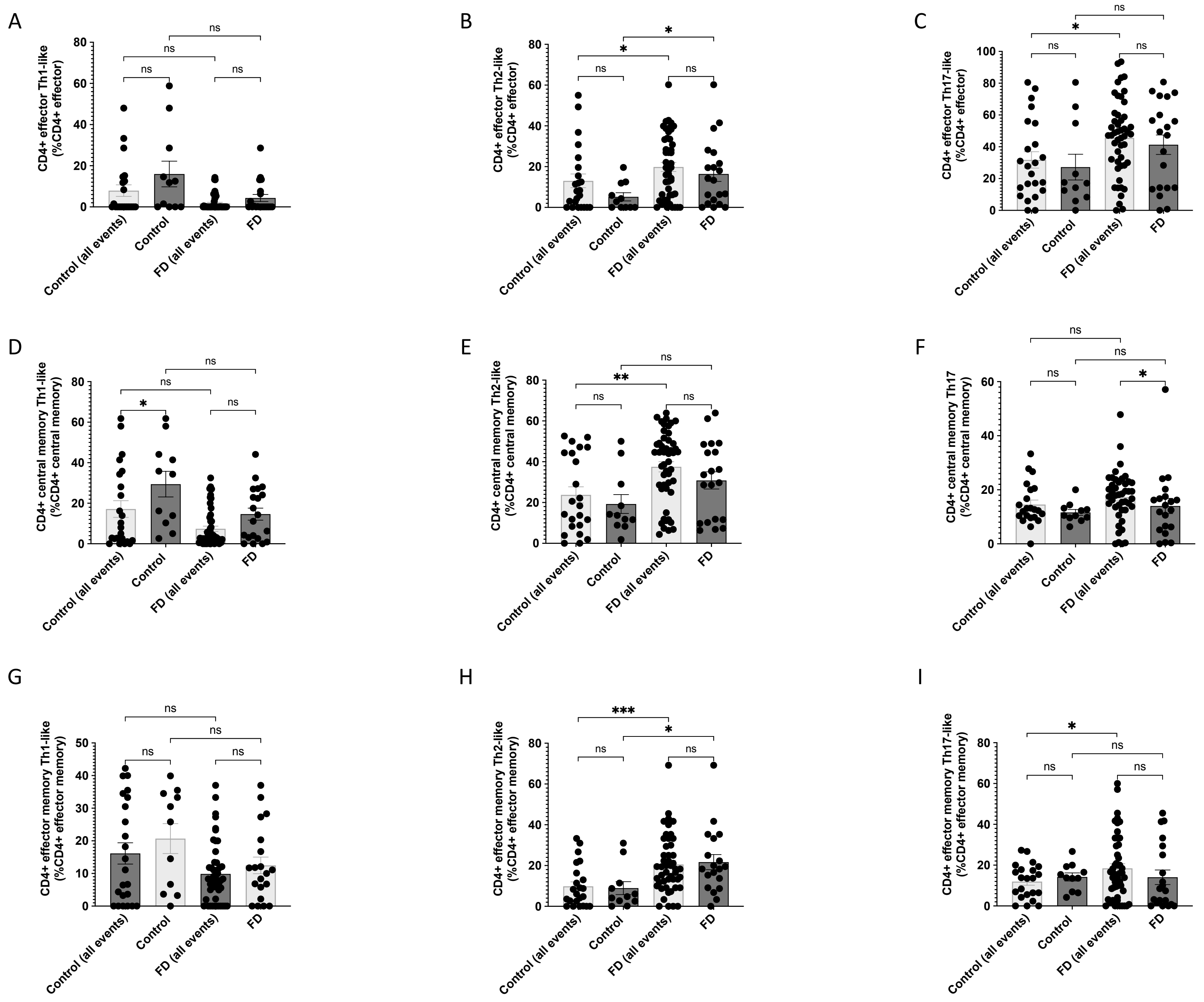

Supplementary Figure 7: Sensitivity analysis of mucosal samples with the maximum event number recorded compared to the entire FD and control cohorts.

Effector (A) Th1, (B) Th2, (C) Th17; central memory (D) Th1, (E) Th2, (F) Th17 and effector memory (G) Th1, (H) Th2, (I) Th17 –like populations in duodenal samples with the maximum event number recorded (‘all events’) were compared to the total cohort to test if the low event number in some samples may contribute to the findings. Data presented as mean±SEM. Statistical analysis: (A, B, C, D, E, F, G, H) Non-parametric ANOVA, (I) parametric on—way ANOVA. *$p<0.05$, **$p<0.01$, ***$p<0.001$.

Supplementary Figure 8

A B

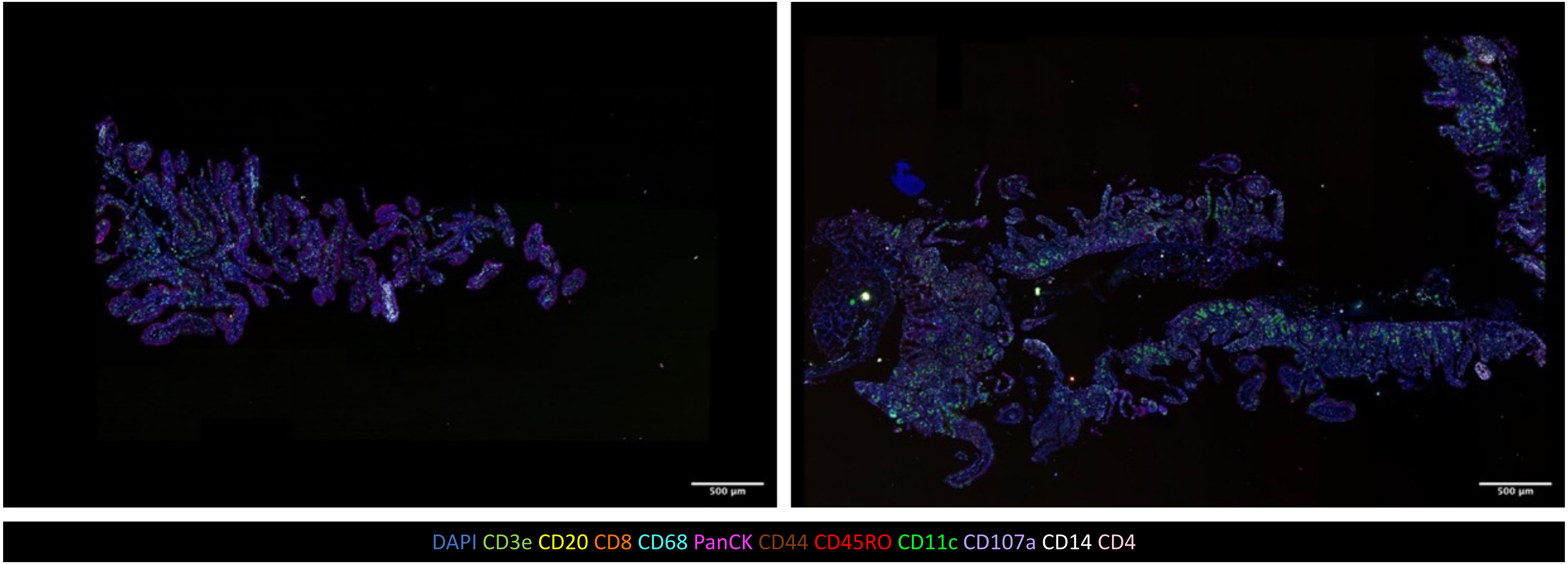

Scale bar = 500µM, 2.15x zoom

C

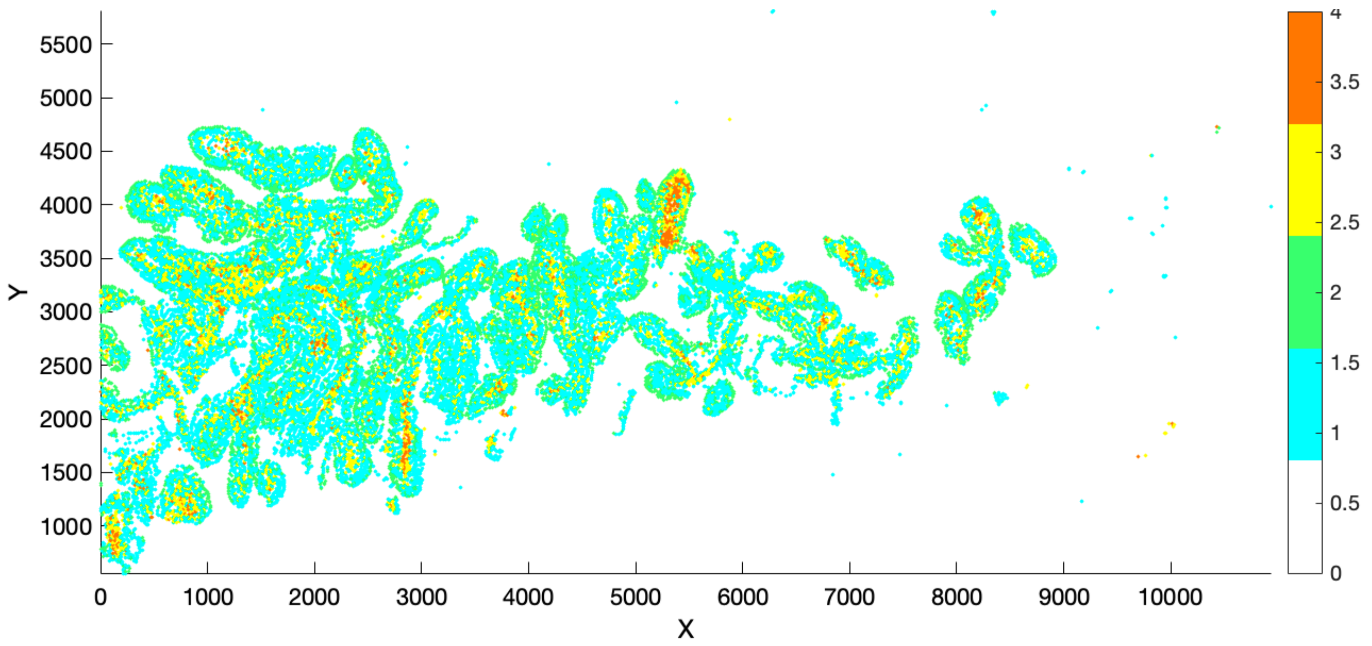
5500
5000
4500
4000
3500
3000
2500
2000
1500
1000
Y
0
1000
2000
3000
4000
5000
6000
7000
8000
9000
10000
X
4
3.5
3
2.5
2
1.5
1
0.5
0

D

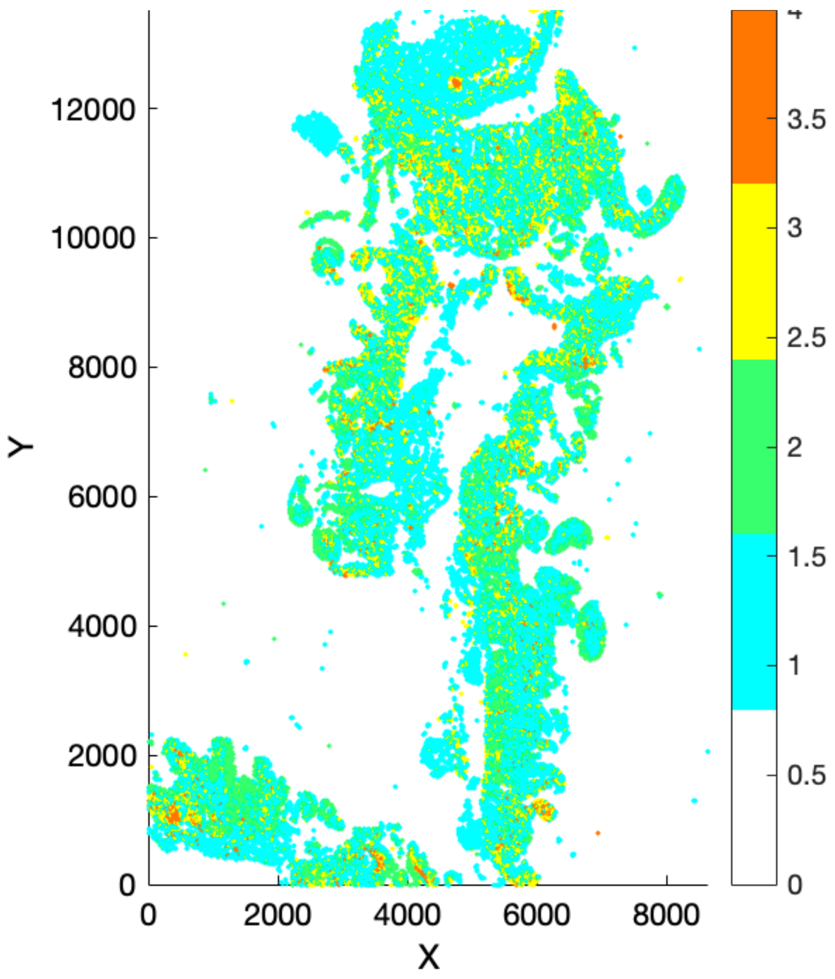
12000
10000
8000
6000
4000
2000
0
Y
0
2000
4000
6000
8000
X
4
3.5
3
2.5
2
1.5
1
0.5
0

Supplementary Figure 8: Duodenal biopsies analysed using Akoya Phenocycler platform.

Images are to demonstrate the size and general structure of the biopsies used for the (A) control and (B) FD patient. Scale bar = 500μM, images taken at 2.15x zoom. CytoMAP was used to algorhymically cluster cells into regions with similarities and a graphical representation of the distribution of these regions throughout the biopsies is provided for the (C) control and (D) FD sample.

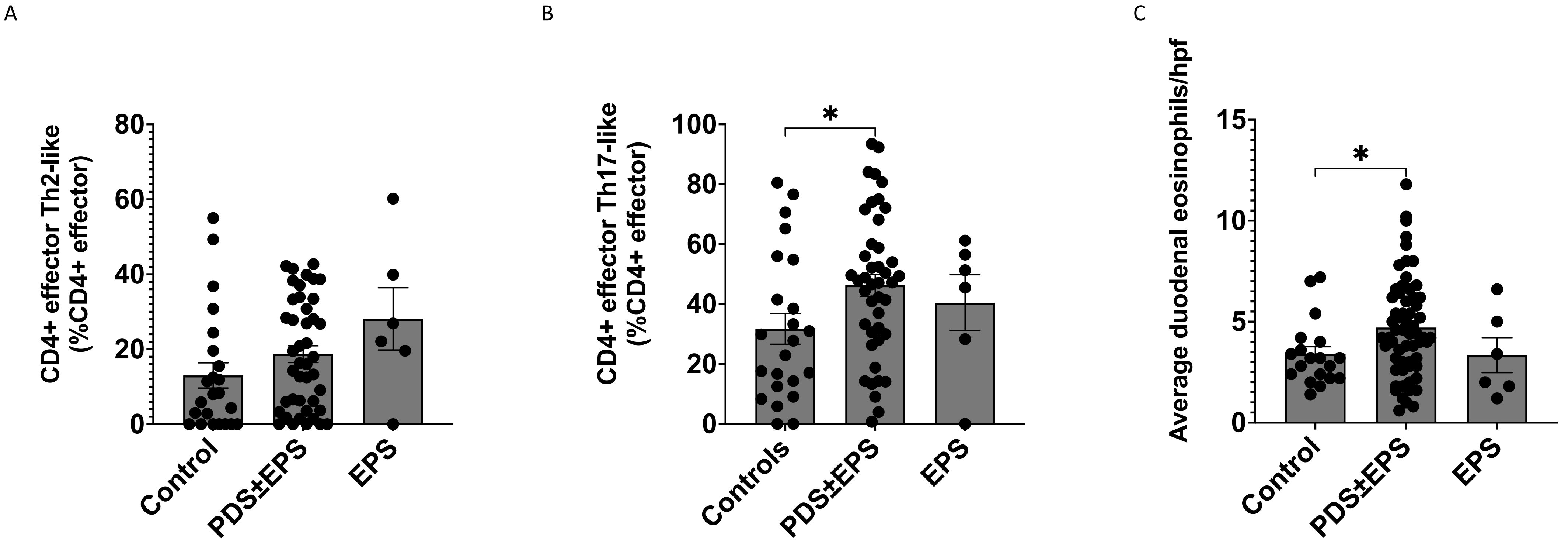
A
CD4+ effector Th2-like (%CD4+ effector)
0
20
40
60
80
Control
PDS±EPS
EPS
B
CD4+ effector Th17-like (%CD4+ effector)
0
20
40
60
80
100
*
Controls
PDS±EPS
EPS
C
Average duodenal eosinophils/hpf
0
5
10
15
*
Control
PDS±EPS
EPS

Supplementary Figure 9: Salient findings in FD separated by meal-associated symptoms compared to epigastric symptoms only.

The (A) $CD4^+$ effector Th2-like, (B) effector Th17-like and (C) duodenal eosinophil numbers were investigated in FD patients classified by those with (n=43) and without (n=6) meal associated symptoms, compared to those with epigastric pain only. n=12 controls, n=32 for FD. Data presented as mean±SEM. Statistical analysis: Non-parametric ANOVA. *p<0.05.
